# Value co-creation and customer satisfaction in last-mile delivery services: The roles of digital accessibility and delivery service quality

**DOI:** 10.1371/journal.pone.0359308

**Published:** 2026-10-06

**Authors:** Wanyang Zhou, Leo D. Manansala, Alice Valerio, Xiaoting Sun

**Affiliations:** 1 School of Tourism and E-Commerce, Baise University, Guangxi Zhuang Autonomous Region, China; 2 College of Professional and Graduate Studies, De La Salle University-Dasmariñas, Dasmariñas City, Philippines; 3 School of Finance and Economics, Henan Polytechnic University, Jiaozuo, China; Dong-A University College of Business Administration, KOREA, REPUBLIC OF

## Abstract

Despite substantial improvements in speed and coverage, customer satisfaction with last-mile delivery remains inconsistent, particularly in complex urban environments where rising consumer expectations, traffic congestion, and operational pressures create persistent challenges. This study investigates the influence of customer value co-creation behaviors, customer participation behaviors (CPB), and customer citizenship behaviors (CCB), on customer satisfaction (CS), examining the mediating role of delivery service quality (DSQ) dimensions and the moderating role of digital accessibility (DA). Data were collected from 426 urban consumers in China, exceeding the minimum sample required by Cochran’s formula. Structural equation modeling (SEM) was used to test direct, mediating, and moderating effects. Findings reveal that CPB directly enhances satisfaction, whereas CCB primarily affects satisfaction indirectly through operational, customization, and personal contact quality. Digital accessibility strengthens the effects of CPB and CCB on DSQ but has a limited direct influence on satisfaction. The study’s novelty lies in simultaneously examining participation and citizenship behaviors, multi-dimensional service quality, and digital accessibility in the context of urban last-mile delivery, a combination not previously explored. Practically, the findings provide actionable guidance for logistics firms to integrate customer engagement strategies with accessible digital platforms to optimize service quality and enhance satisfaction.

## Introduction

The fast growth of e-commerce has driven last-mile delivery into a competitive space where service innovation, rather than just delivery speed, is critical. China represents a prominent example of this dynamic. Its e-commerce industry is growing rapidly: the market will increase by 11.9 percent in 2023, reaching nearly USD 2.2 trillion, with an estimated compound annual growth rate of 9.9 percent from 2024 to 2028 [1]. This growth is supported by investments in logistics infrastructure, digital platforms, and national delivery systems, allowing for next-day or same-day delivery for much of the population [2, 3]. But rapid delivery and expansive reach have not always translated into satisfaction in these processes, with urban settings experiencing frustration points caused by consumer demand, traffic issues, labor shortages, and operational challenges [4, 5].

Much of the prior literature addresses these pain points from an inward-looking perspective, focusing on infrastructure and operational improvements to advance logistics efficiency, capacity, and service quality [6–8]. These improvements are important; however, they do not completely solve the issue. Operational efficiency does not thoroughly account for the variations in satisfaction, even among advanced delivery networks. This issue has allowed for other approaches to emerge that go beyond firm-centered processes.

One emerging approach has highlighted that last-mile delivery is an interactive process, defined by engagement activities between delivery personnel and consumers [9, 10]. Consumers now commonly engage in co-creating value by scheduling delivery appointments, providing guidance, using smart delivery applications, and giving feedback throughout the process. This involvement is analogous to the process of customer value co-creation (CVC), which views consumers as doing more to shape service value than simply receiving it [11]. Research from the hospitality, tourism, e-services, banking, and healthcare industries has shown that CVC can enhance satisfaction by improving relational, interactive, and experiential aspects of services [12–15]. Feedback-providing, information-giving, and direct interaction with couriers have already become commonplace avenues occupied by co-creation in last-mile delivery; understanding how these factors drive service quality and satisfaction through these mechanisms can illuminate the challenges faced by providers balancing operational concerns with customer experience.

Delivery service quality (DSQ) remains a primary determinant of logistics satisfaction, measured through three dimensions: personal contact quality, customization quality, and operational quality [8, 16, 17]. Personal contact quality reflects interactions with delivery personnel and their role in shaping perceptions of empathy, professionalism, and responsiveness. Customization quality is the extent to which services are tailored for particular needs, usually utilizing digital applications that enable delivery options for specific orders and firm differentiation [18, 19]. Operational quality is about the accuracy, timeliness, and condition of parcels upon delivery; these are views that tie into the larger impressions of reliability and efficiency [8, 20, 21]. While these dimensions are relatively established, little prior research addresses how CVC behaviors drive each dimension and mediate the link between co-creation and satisfaction. Research from related service settings suggests that active consumers can compensate for errors, improve linkages, and build rapport [22, 23]. It remains underexamined whether operational improvements function in last-mile delivery, constituting an obvious gap in the literature.

The digitization of logistics also brings greater salience to customer engagement. Digital accessibility, defined as the quality of allowing consumers to engage providers through digital platforms, may further allow or enable the use co-creation by controlling consumers’ communicative, instructive, or integrative abilities [24, 25]. From the standpoint of dynamic capabilities, digital accessibility heightens a firm’s resources’ ability to integrate customer assets into service processes to enhance service quality and satisfaction. Mobile telematics and Internet of Things (IoT) systems strengthen these capabilities by enabling efficient information sharing, real-time order tracking, risk management, and stakeholder coordination [26]. Digital service design, focusing on customer control, participation, and customization, is also a key enabler of co-creation, with its ability to build more personalized experiences, perceptions of agency, and satisfaction [27]. However, prior research has been reluctant to use digital accessibility as a moderator throughout the co-creation process. Without this approach, it remains questionable whether digital accessibility strengthens or inhibits the impact of CVC on delivery service quality and satisfaction.

CVC itself involves two interrelated but distinct behavioral dimensions: customer participation behavior and customer citizenship behavior [28]. Participation behavior is tied to direct assistance or improvements in service provision, such as scheduling delivery or helping personnel. Citizenship behavior consists of extra-role behaviors, such as referral or assistance to other consumers [29]. Although prior literature in other service industries indicates both of these dimensions positively contribute to satisfaction, their impacts and processes in last-mile delivery are underdeveloped [23, 30]. Evidence from Tokyo indicates that customer participation can uplift operational burdens and enhance delivery effectiveness [22]. However, direct empirical testing of participation and citizenship in shaping delivery service quality and satisfaction remains untested in a logistics context. This gap must be addressed so that customer co-creation can lead to positive service outcomes.

Customer engagement through processual and operational dimensions can be further strengthened through institutional and service design differences. While prior literature highlights customer engagement, it has yet to explore how these organizational architectures drive the impacts of CVC behaviors on delivery service quality and satisfaction. Understanding this facilitatory role can provide important insight into the evolving relationship between operations and consumer experience in last-mile delivery within the logistics sector.

Looking at all these gaps together, the suggested framework explains the influence of CVC on customer satisfaction in last-mile delivery through delivery service quality as a mediator and digital accessibility as a moderator. Hence, this study aims to investigate how customer participation and citizenship behaviors influence satisfaction, the mediating effects of the key dimensions of service quality, and how the conditions are moderated by digital accessibility. The study integrates both behavioral and technological perspectives to clarify the mechanisms through which active customer involvement results in satisfaction in digitally enabled logistics ecosystems.

The study focuses on China’s rapidly expanding urban logistics setting and contributes to theory and practice. First, it extends Service-Dominant Logic and dynamic capabilities theory concerning last-mile delivery, providing insight into the processes of interaction through which service value and satisfaction are manifested. Second, it guides logistics firms to enhance service quality, engage customers for active understanding, and use digital platforms to gain operational efficiency and satisfaction. The study is novel as it examines customer participation and citizenship conduct; the mediating role of the dimensions of delivery service quality; and the moderating influence of digital accessibility, reconciling the behavioral and technological views of value co-creation.

While prior works typically stress smooth logistics and quality of service from the provider’s vision, this research puts customers at the center of value creation. By integrating customer behavior with service quality and digital accessibility, it provides innovative insights for firms that thrive in increasingly competitive logistics markets.

### Literature review and hypotheses development

A growing body of last-mile delivery research has converged to a common message: Customer satisfaction is not the product of technology, service quality, or customer behavior in isolation, but rather the consequence of their synergy. Digital transformation, customer value co-creation, service quality, and digital accessibility act as a holistic system rather than individual drivers. Many streams of the literature continue to address these topics discretely. Empirical evidence integrating participation and citizenship behaviors, multi-faceted service quality, and digital accessibility as an integrated approach remains scarce. To address this limitation, we investigate the interplay of behavioral engagement and technological conditions on satisfaction in contemporary logistics contexts where customers are no longer mere recipients but contributors to the service outcome.

### Digital transformation of logistics companies

For logistics firms operating in e-commerce settings, digital transformation is no longer an issue of strategic prerogative but a functional imperative. More stringent expectations for speed, flexibility, and reliability have propelled companies to integrate the Internet of Things, mobile telematics, and predictive analytics into their operations [31–34]. These instruments boost visibility, improve responsiveness to demand variability, and foster coordination with supply chain partners, which is particularly important in the last-mile segment [35].

What often gets overshadowed is the fact that digital transformation permeates the customer role as well. Platforms dedicated to tracking, evaluation, and service definition create new avenues for interaction and collaboration, thus encompassing customers in the value production process [36]. Firms with higher digital maturity levels are therefore better positioned not only to streamline the operational process but also to facilitate customer involvement. The joint operational and relational role of technology underscores why digital transformation needs to be treated in conjunction with service design and customer engagement in last-mile delivery.

### Customer value co-creation behavior and customer satisfaction

CVC captures the notion that consumers shape service outcomes through active collaboration and interaction with service providers [13, 14]. Yi and Gong [28] provoke an insightful distinction between customer participation behaviors and customer citizenship behaviors. Whereas participation behaviors are instrumental and expected, including providing information or responding to a scheduling request, citizenship behaviors transcend formal roles, are voluntary, and support other customers [29].

Participation behaviors contribute directly to service efficiency by decreasing ambiguity, enabling co-production, and value-in-use [37–39]. Citizenship behaviors are leveraged more indirectly, helping to build brand image, social value, and relational ties, for example, through advocacy and online engagement, and are motivated by emotional proximity [40–42].

However, emerging studies also caution against assuming that co-creation is universally beneficial. Customer participation may increase perceived effort [43], shift operational responsibilities from firms to consumers [44, 45], and generate resistance when engagement demands [46] exceed perceived value-for-money. In logistics contexts, customers often prioritize convenience, such as door-to-door delivery, over active involvement in service processes. When co-production mechanisms (e.g., parcel lockers, self-scheduling, or digital confirmations) are perceived as cost-saving measures that primarily benefit firms, customers may exhibit minimal engagement or even dissatisfaction [47, 48]. This “dark side” perspective suggests that co-creation effectiveness is contingent upon perceived fairness, convenience, and effort–benefit balance.

In logistics contexts, service quality and trust invariably determine loyalty and satisfaction. Dependable delivery, clear communication, accurate information, and a seamless process all help to create evaluative criteria for customers [49–51]. Trust compounds satisfaction by providing legitimacy and credibility, such that customers remain loyal despite competitive pressure [52]. Loyal relationships are also not entirely homogeneous. For instance, in environments with scarce options, satisfaction persists regardless of actual service quality, indicating a decoupling of contextual factors.

Customer satisfaction in last-mile delivery reflects not only effective performance and the meeting of expectations but also emotional response and trust [6]. Empirical evidence indicates that participation behaviors such as responsive actions and accurate information sharing allow providers to frame services more effectively, resulting in higher satisfaction [4, 53]. Citizenship behaviors then contribute by fostering positive experiences with couriers and strengthening relational trust [54]. Taken together, while co-creation behaviors can enhance efficiency and relational value, their impact on satisfaction depends on whether customers perceive engagement as facilitative rather than burdensome. In combination, these dynamics indicate that customer involvement meaningfully shapes efficient, personalized, and positive emotional delivery experiences. Hence, we hypothesize the following:

*H1: Customer participation behaviors influence customer satisfaction.*
*H2: Customer citizenship behaviors influence customer satisfaction.*

### Customer value co-creation behavior and last-mile delivery service quality

The quality of last-mile delivery service is therefore a primary driver for satisfaction and is usually viewed through three lenses: personal contact quality, operational quality, and customization quality [8]. Personal contact quality captures the personnel interaction and includes politeness, empathy, and the ability to address problems [55, 56]. Operational quality refers to dependability, accuracy, punctuality, and the handling of goods [57, 58]. Customization quality refers to the degree of individualization of services offered, such as a flexible delivery window or special instructions [59, 60].

According to prior work, participation and citizenship behaviors improve the qualitative markers. Accurate instructions and prompt responses increase dependability, while feedback and advocacy help foster personalized contact and customization [61, 62]. In digital contexts, participation deepens co-creation by facilitating co-production and value-in-use, for instance, catalyzing citizenship behaviors [39, 63]. Organizational support and fairness also facilitate engagement [64].

That said, the impacts of behaviors are uneven across contexts. Collectivism, for instance, may reinforce or diminish interpretive cues, whereas customer-to-customer interaction does not always lead to citizenship behaviors [65, 66]. This indicates that despite improvements in service quality, the social and cultural context also shapes the realized impact. Drawing from empirical insights, we propose the following:

*H3: Customer participation behaviors influence last-mile delivery service quality.*
*H4: Customer citizenship behaviors influence last-mile delivery service quality.*

### Last-mile delivery service quality as a mediating factor

Customer participation and citizenship behaviors have a direct effect on satisfaction, but a more indirect issue is how these effects are realized in terms of service quality. Service-Dominant Logic conceptualizes value as the result of resource integration and collaborative actors’ experiences [11]. From this perspective, customers’ inputs lead to higher operational effectiveness for personalization and interpersonal mechanisms, which contribute to the improvement of satisfaction. Participation behaviors tend to improve reliability and customization, while citizenship behaviors increase employees’ personal contact and relational trust, and the mediating mechanism in which both effects happen is service quality [67, 68].

Hospitality showed that interactive and personalized service features, such as employees’ responsiveness and comfort-related characteristics, contributed to higher emotional value and at least double satisfaction [69]. Studies in AI-enabled services showed that with the integration of automated processes with human involvement, they deliver a more integrated experience and satisfaction due to better empathy and intercommunication [70].

Logistics and service operations also see service quality as a mediator, given the growing amount of evidence [57, 62]. Operational coordination, personalization, and cooperative contact are the mechanisms through which engagement translates into satisfaction. When those elements align with customer expectations, satisfaction, trust, and retention would occur, given the alignment, but threats of data privacy and overpersonalization need to be considered [68]. Such findings support the following hypotheses:

*H5: Delivery service quality mediates the relationship between customer participation behaviors and customer satisfaction.*
*H6: Delivery service quality mediates the relationship between customer citizenship behaviors and customer satisfaction.*

### Digital accessibility as a moderating factor

Digital accessibility is usually characterized by the ease of customers’ access and utilization of digital platforms, involving aspects of usability, availability, and ease attributes [71, 72]. Such greater accessibility would facilitate patients’ experiencing lower entry barriers and experience smooth participation in co-creation actions [23]. The Technology Acceptance Model states that considering digital tools useful and easy to use would increase engagement and satisfaction [23].

In last-mile delivery, such as real-time tracking, mobile, and interactive platforms, they would facilitate personalization, better communication with couriers, and enhance the effects on service quality and satisfaction [73, 74]. Real-time tracking would increase transparency and trust from lower-operational uncertainty, while interactive platforms would allow a two-way channel and personalized experiences with service [75, 76].

Still, the empirical evidence is nuanced. Studies reveal that digital accessibility may potentially bring incremental value to the contributions of participation and citizenship behaviors, while it also may constitute a distraction in cases when platforms are complex and intrusive [74]. Cultural variables and C2C relations make such a complex as well [65, 66]. Such contradictions imply that the impacts of digital accessibility would be contingent on the design of quality and the broad social context. Based on such evidence, the following hypotheses are postulated:

*H7: Digital accessibility moderates the relationship between customer participation behaviors and delivery service quality.*
*H8: Digital accessibility moderates the relationship between customer citizenship behaviors and delivery service quality.*
*H9: Digital accessibility moderates the relationship between customer participation behaviors and customer satisfaction.*
*H10: Digital accessibility moderates the relationship between customer citizenship behaviors and customer satisfaction.*

Fig 1 illustrates all hypothesized relationships, including direct, mediating, and moderating effects. The diagram clearly presents CPB and CCB as antecedents, DSQ dimensions as mediators, digital accessibility as a moderator, and customer satisfaction as the outcome variable.

**Fig 1:**
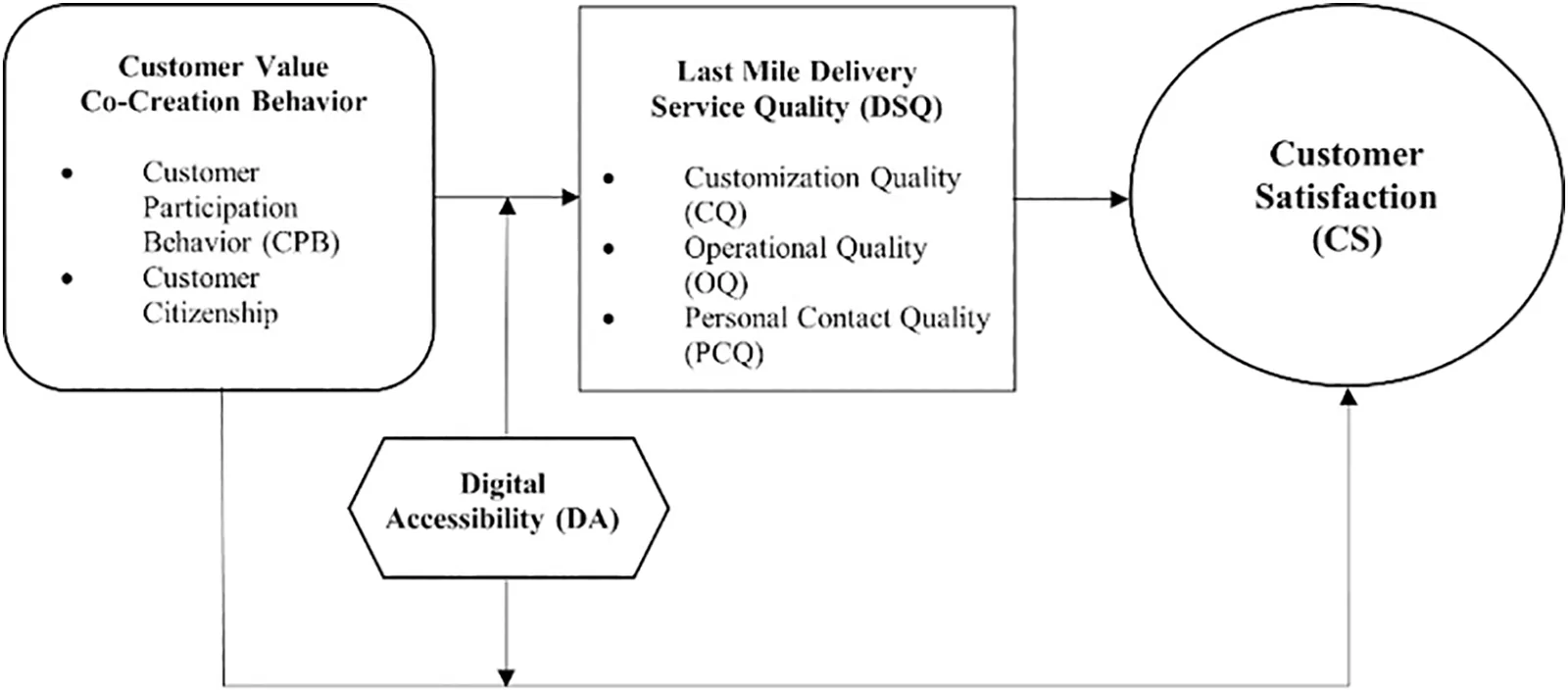
Conceptual framework of the study.

## Methodology

### Sources of data

Data were collected from 426 consumers of last-mile delivery services in Nanning, China, from March 15 to June 28, 2025, using a structured self-administered questionnaire. Nanning, the capital of Guangxi Zhuang Autonomous Region, is a rapidly urbanizing city with over 8 million residents and a thriving digital economy. The city features diverse residential communities and multiple logistics providers [77, 78], making it a representative setting for examining last-mile delivery service experiences.

The minimum required sample, calculated via Cochran’s formula at a 95% confidence level and 5% margin of error, was 385 respondents [79], so the actual sample exceeds this threshold. Stratified sampling ensured proportional representation across Nanning’s administrative districts. Table 1 presents the respondent profile.

**Table 1:** Socio-demographic profile of last-mile delivery service customers, China, 2025.

Characteristic	Frequency	Percent (%)
*Age (years)*		
20–25	162	38.0
26–30	104	24.4
31–35	56	13.10
36–40	34	8.00
41–45	42	9.90
46–50	20	4.70
51–55	6	1.40
56–60	2	0.50
Mean	30.03	
SD	9.10	
Minimum	20.00	
Maximum	60.00	
*Gender*		
Female	254	59.62
Male	172	40.38
Total	426	
*Years of schooling*		
**7**–10	9	2.1
11–14	52	12.2
15–18	280	65.7
19–22	85	20.0
Mean	16.04	
SD	2.53	
Minimum	7	
Maximum	22.00	
*Net monthly income (RMB)*		
2,000–4,399	89	20.90
4,400–6,799	141	33.10
6,800–9,199	142	33.30
9,200–11,599	49	11.50
11,600–13,999	5	1.20
Mean	5,148.59	
SD	2,271.39	
Minimum	2,000.00	
Maximum	1,3000	

Data were collected through online surveys and telephone interviews. Respondents were recruited through partnerships with major logistics companies, where delivery personnel distributed QR-coded survey links via parcel lockers and package deliveries. Additional participants were reached through WeChat announcements. The questionnaire measured customer participation and citizenship behaviors, service quality, digital accessibility, and customer satisfaction. A pilot test with 30 logistics management students refined questionnaire clarity and structure.

### Ethical approval and participant consent

Ethical approval for this study was granted by the De La Salle University–Dasmariñas Research Ethics Committee before data collection. Informed consent was obtained electronically from all participants through an online consent form presented at the beginning of the survey, with consent indicated by selecting an agreement option. All participants were adults (18 years and above), and no minors were involved. No waiver of informed consent was granted.

### Research instruments

A structured questionnaire to measure constructs related to customer value co-creation behaviors, last-mile delivery service quality, digital accessibility, and customer satisfaction was used. A five-point Likert scale was used for all items, where 1 = “very low” and 5 = “very high.” Table 2 presents the conceptual constructs, indicators, and sources for consumer participation behavior, customer citizenship behavior, service quality dimensions, satisfaction, and digital accessibility.

**Table 2:** Construct measures of consumer value co-creation behaviors and their outcomes in last-mile delivery services.

Construct	Indicators	References
*Independent Variables*		
Customer Participation Behavior (CPB)	Information sharing, responsible behavior, and personal interaction	Yi & Gong (2021)
Customer Citizenship Behavior (CCB)	Feedback, Advocacy, Helping, and Tolerance	Yi & Gong (2021)
*Dependent Variable*		
Customer Satisfaction (CS)	Satisfaction with performance, Service meeting expectations, Evaluation of service quality, Overall satisfaction, Positive emotions, Satisfaction with choice	Lin et al. (2023)
*Mediating Variable*		
Last Mile Delivery Service Quality (DSQ)		
• Customization Quality (CQ)	Service meeting customer requests, Diverse service options, Meeting specific needs, Unique services, Unmatched customized services	Lin et al. (2023)
• Operational Quality (OQ)	Accurate product delivery, Adequate storage capacity, Reliable service execution, Goods intact, Efficient document processing	Lin et al. (2023)
• Personal Contact Quality (PCQ)	Empathy, Problem-solving ability, Expertise, Positive attitude, Politeness	Lin et al. (2023)
*Moderating Variable*		
Digital Accessibility (DA)	Ease of accessing digital services, Simplicity of using digital services, Availability of digital services, Reliable last-mile digital services	Hien (2024)

The questionnaire was organized into four sections. The first section collected demographic information, including age, gender, education, income, and region, to allow comparative analyses of participation behaviors and satisfaction across groups. The second section assessed customer value co-creation behaviors. Participation behaviors included information sharing, responsible behavior, and personal interaction, while citizenship behaviors encompassed feedback, advocacy, helping, and tolerance [28].

The third section evaluated delivery service quality outcomes—personal contact quality, operational quality, and customization quality—and consumer satisfaction, including performance satisfaction, expectation fulfillment, evaluation of service quality, overall satisfaction, positive emotions, and satisfaction with service choice [8]. The final section measured digital accessibility, focusing on ease of access, simplicity of use, and availability of digital services [71]. These dimensions collectively provide a comprehensive framework for analyzing the relationships among co-creation behaviors, service quality, digital accessibility, and customer satisfaction in last-mile logistics.

### Model specification

To test the proposed hypotheses, Partial Least Squares Structural Equation Modeling (PLS-SEM) was employed, which allows for the simultaneous estimation of multiple dependent variables and mediating effects. PLS-SEM is particularly suitable for this study due to its flexibility with non-normal data, exploratory focus, and capacity to handle complex models involving multiple latent constructs [80, 81].

The structural model specifies the direct effects of customer value co-creation behaviors—CPB and CCB, on delivery service quality (DSQ) and customer satisfaction (CS). Hypotheses H1 and H2, which posit that CPB and CCB influence CS, are tested through the following structural equation:


$$\begin{array}{c} CS\;=\;\beta_{\text{3}}CPB\;+\;\beta_{\text{4}}CCB\;+\varepsilon_{\text{2}} \end{array}$$

Similarly, H3 and H4, which examine the effects of CPB and CCB on DSQ, are evaluated via the direct paths:


$$\begin{array}{c} DSQ\;=\;\beta_{\text{1}}CPB\;+\;\beta_{\text{2}}CCB\;+\varepsilon_{\text{1}} \end{array}$$

Where $\beta_{\text{1}}$to $\beta_{\text{4}}$ are path coefficients indicating the strength of relationships, and $\varepsilon_{\text{1}}$ and $\varepsilon_{\text{2}}$ are the respective error terms.

PLS-SEM also supports the examination of complex relationships, such as mediating and moderating effects. This feature allows the model to capture how CPB and CCB indirectly affect customer satisfaction through delivery service quality, and how digital accessibility conditions the strength of these relationships. By estimating these paths simultaneously, PLS-SEM provides a robust framework to assess the hypothesized effects and their significance within the digitized last-mile delivery context.

DSQ is hypothesized to mediate the effect of CPB (H5) and CCB (H6) on CS. The mediation is captured through:


$$\begin{array}{c} DSQ\;=\;\alpha_{\text{1}}CPB\;+\;\alpha_{\text{2}}CCB\;+\varepsilon_{\text{3}} \end{array}$$

$$\begin{array}{c} CS\;=\;\gamma DSQ\;+\;\delta_{\text{1}}CPB\;+\;\delta_{\text{2}}CCB\;+\varepsilon_{\text{4}} \end{array}$$

The indirect effect of CPB on CS through DSQ is calculated as ${Indirect}_{CPB}=\;\alpha_{\text{1}}\;x\;\gamma$, and for CCB as ${Indirect}_{CCB}=\;\alpha_{\text{2}}\;x\;\gamma$. The total effect combines direct and indirect paths:


$$\begin{array}{c} {Total}_{CPB}=\;\delta_{\text{1}}\;+\;\left(\alpha_{\text{1}}x\;\gamma \right) \end{array}$$

$$\begin{array}{c} {Total}_{CCB}=\;\delta_{\text{2}}\;+\;(\alpha_{\text{2}}x\;\gamma ) \end{array}$$

Digital accessibility is incorporated as a moderator to examine whether it alters the strength or direction of the relationships between CPB/CCB and delivery service quality (H7–H8), as well as CPB/CCB and CS (H9–H10). Moderation is tested using an interaction term in the structural model:


$$\begin{array}{c} DSQ\;=\;\beta_{\text{1}}CPB\;+\;\beta_{\text{2}}CCB\;+\beta_{\text{5}}(CPB\;x\;DA)\;+\;\beta_{\text{6}}(CCB\;x\;DA)+\varepsilon_{\text{5}} \end{array}$$

$$\begin{array}{c} CS\;=\;\beta_{\text{3}}CPB\;+\;\beta_{\text{4}}CCB\;+\beta_{\text{7}}(CPB\;x\;DA)\;+\;\beta_{\text{8}}(CCB\;x\;DA)+\varepsilon_{\text{6}} \end{array}$$

Significant interaction coefficients, $\beta_{\text{5}}$ to $\beta_{\text{8}}$, indicate that digital accessibility strengthens or weakens the effects of CPB and CCB on delivery service quality and customer satisfaction.

A series of diagnostic tests was also conducted to ensure the reliability and validity of the measurement and structural models. Multicollinearity among constructs was examined using the Variance Inflation Factor (VIF), while common method bias was assessed through Harman’s single-factor test. Factor loadings were evaluated to confirm indicator relevance, and construct reliability and validity were verified using Cronbach’s alpha, composite reliability (CR), and average variance extracted (AVE). Discriminant validity was assessed to ensure that constructs were distinct from one another, and model fit was evaluated through appropriate goodness-of-fit measures, confirming the robustness and adequacy of the PLS-SEM model for hypothesis testing.

## Results and discussions

To contextualize the subsequent structural and moderating tests, ensuring that the relationships explored in the hypotheses are grounded in the observed data patterns, Table 3 presents the descriptive statistics, which reveal that all key variables are in the last-mile delivery service. This offers a foundation for understanding how customer value co-creation behaviors, last-mile delivery service quality, customer satisfaction, and digital accessibility interact.

**Table 3:** Descriptive statistics of key variables in last-mile delivery services.

Variable	Mean	SD	Interpretation
Customer Participation Behavior	3.87	0.422	High
Information seeking	3.91	0.413	High
Information sharing	3.75	0.415	High
Responsible behavior	3.90	0.386	High
Personal interaction	3.92	0.472	High
Customer Citizenship Behavior	3.78	0.780	High
Feedback	3.72	0.847	High
Advocacy	3.63	0.751	High
Helping	3.90	0.671	High
Tolerance	3.86	0.852	High
Service Quality	4.09	0.793	High
Personal contact quality	4.10	0.777	High
Operational quality	4.13	0.782	High
Customization quality	4.05	0.819	High
Customer Satisfaction	3.95	0.898	High
Satisfaction with performance	4.09	0.823	High
Service meeting expectations	3.86	0.854	High
Evaluation of service quality	4.14	0.727	High
Overall satisfaction	3.90	1.027	High
Positive emotions	3.94	0.917	High
Satisfaction with choice	3.93	0.963	High
Digital Accessibility	3.91	0.867	High
Ease of accessing digital services	3.86	0.876	High
Simplicity of using digital services	3.98	0.855	High
Availability of digital services	3.92	0.875	High
Reliable last-mile digital services	3.87	0.862	High

Table 3 shows that all key variables in last-mile delivery services are rated high by respondents. Customer value co-creation behaviors, including participation and citizenship, indicate active engagement through information sharing, personal interaction, and voluntary contributions, with low variability in participation and higher variability in certain citizenship behaviors. Service quality—personal contact, operational, and customization—also scores high, though operational and customization show slight differences due to infrastructure and service personalization. Customer satisfaction is generally high, with moderate variability in expectations and overall satisfaction, highlighting areas for improvement. Digital accessibility is positively rated across ease of access, simplicity, availability, and reliability, emphasizing the role of digital platforms in supporting value co-creation and satisfaction. These results provide a solid basis for testing the study’s hypotheses.

### Measurement model assessment

Consistent with the two-step SEM approach recommended by Hair et al. [80], the analysis began with the assessment of the measurement model to evaluate the reliability and validity of the constructs. Once the measurement model was confirmed, the structural model was examined to test the hypothesized relationships among the latent variables. Before hypothesis testing, several diagnostic tests were conducted to ensure the robustness of the model.

Table 4 presents the VIF values for the inner model. All VIF values are below 5, indicating that multicollinearity among latent variables is not a concern [80].

**Table 4:** VIF values of inner model.

Inner Model Path	VIF
CCB → CQ	2.033
CCB → CS	2.510
CCB → OQ	2.033
CCB → PCQ	2.033
CPB → CQ	2.781
CPB → CS	3.052
CPB → OQ	2.781
CPB → PCQ	2.781
CQ → CS	3.137
DA → CQ	1.669
DA → CS	2.219
DA → OQ	1.669
DA → PCQ	1.669
OQ → CS	1.966
PCQ → CS	2.719

The results of Harman’s single-factor test (Table 5) indicate that the first factor accounts for 44.92% of the total variance, which is below the 50% threshold commonly used to signal common method bias. This suggests that the measurement data are not significantly affected by common method variance, providing confidence in the reliability of the constructs and the validity of subsequent PLS-SEM analyses.

**Table 5:** Harman’s single-factor test results.

Component	Initial Eigenvalues	% of Variance	Cumulative %	Rotation Sum of Squared Loadings	% of Variance	Cumulative %
1	24.255	44.916	44.916	24.255	44.916	44.916
2	4.538	8.403	53.319	4.538	8.403	53.319
3	3.094	5.729	59.048	3.094	5.729	59.048
4	2.030	3.759	62.807	2.030	3.759	62.807
5	1.849	3.424	66.232	1.849	3.424	66.232
6	1.324	2.452	68.683	1.324	2.452	68.683
7	1.210	2.242	70.925	1.210	2.242	70.925
8	1.151	2.131	73.056	1.151	2.131	73.056
9	1.019	1.886	74.943	1.019	1.886	74.943
10	0.779	1.443	76.386	–	–	–
11	0.674	1.249	77.635	–	–	–
12	0.595	1.102	78.737	–	–	–
13	0.569	1.054	79.790	–	–	–
14	0.525	0.972	80.763	–	–	–
15	0.492	0.911	81.674	–	–	–
16	0.478	0.886	82.560	–	–	–
17	0.465	0.862	83.421	–	–	–
18	0.440	0.815	84.237	–	–	–
19	0.409	0.758	84.995	–	–	–
20	0.406	0.751	85.746	–	–	–
21	0.389	0.721	86.466	–	–	–
22	0.369	0.683	87.149	–	–	–
23	0.365	0.675	87.824	–	–	–
24	0.354	0.656	88.480	–	–	–

Table 6 presents factor loadings, Cronbach’s alpha, composite reliability (ρa and ρc), and AVE for all constructs. Factor loadings above 0.70 and AVE above 0.50 indicate strong convergent validity. Cronbach’s alpha and composite reliability values exceeding 0.70 confirm internal consistency.

**Table 6:** Convergent validity and internal consistency of measurement constructs.

Variable	Loading Range	Cronbach’s α	ρa	ρc	AVE
CPB	0.624–0.878	0.954	0.955	0.960	0.650
CCB	0.565–0.831	0.916	0.937	0.927	0.520
CQ	0.853–0.915	0.928	0.929	0.946	0.777
PCQ	0.851–0.885	0.922	0.923	0.942	0.763
OQ	0.855–0.907	0.933	0.935	0.949	0.788
CS	0.680–0.899	0.923	0.928	0.941	0.726
DA	0.829–0.862	0.864	0.867	0.908	0.711

Fig 2 presents the factor loadings of all observed variables. Loadings closer to 1 indicate strong associations with their respective latent constructs, confirming that items adequately measure their intended constructs. Items with loadings below 0.50 were removed to improve model fit.

**Fig 2:**
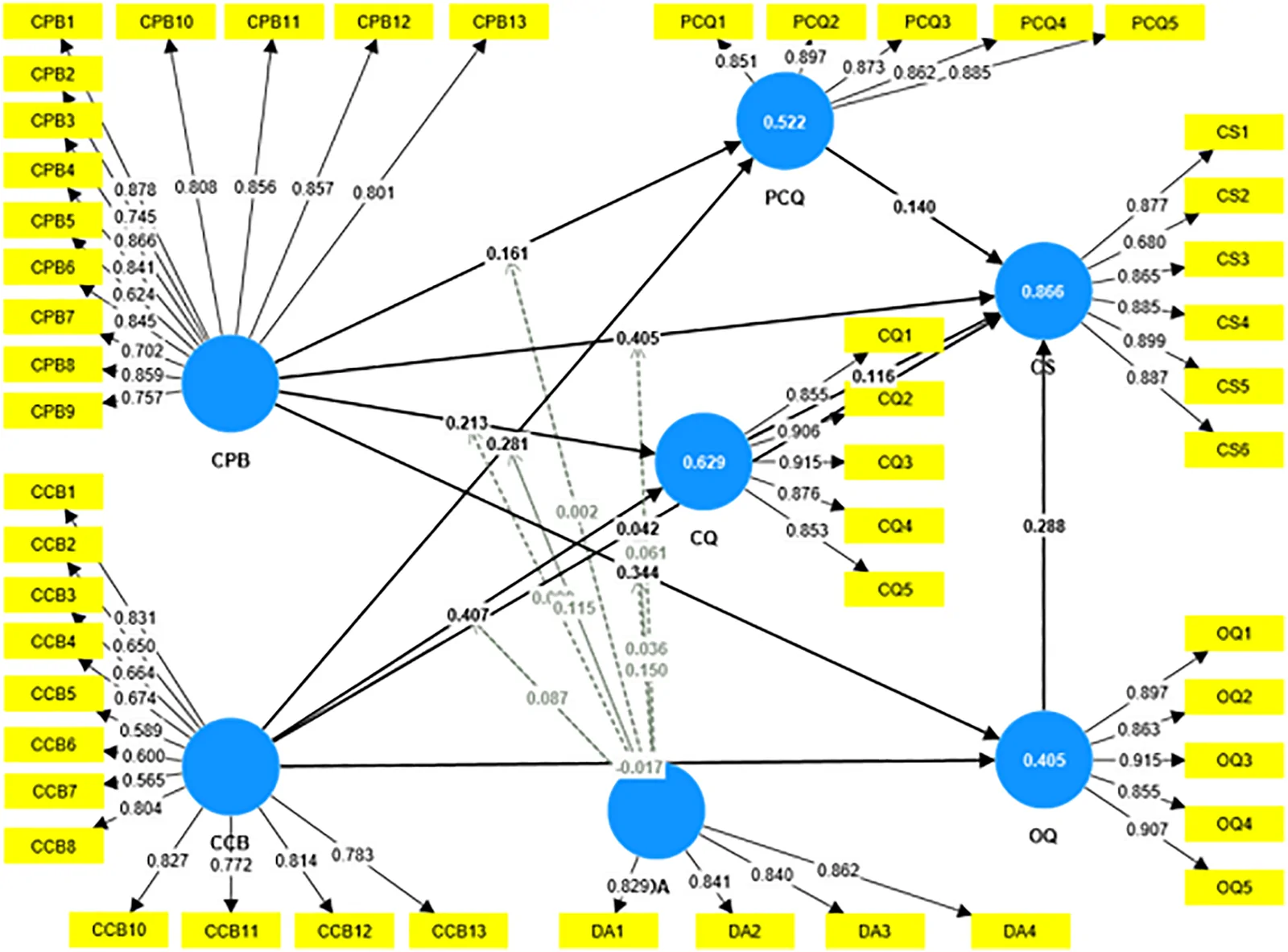
Factor loadings of the constructs of the model in the study.

Table 7 shows the square roots of AVE on the diagonal and inter-construct correlations off-diagonal. Discriminant validity is established as each construct’s square root of AVE exceeds its correlations with other constructs.

**Table 7:** Discriminant validity assessment using the fornell–larcker criterion.

	CCB	CPB	CQ	CS	DA	OQ	PCQ
CCB	0.721						
CPB	0.627	0.806					
CQ	0.721	0.595	0.882				
CS	0.704	0.737	0.767	0.852			
DA	0.601	0.528	0.668	0.746	0.843		
OQ	0.507	0.470	0.578	0.761	0.558	0.888	
PCQ	0.614	0.505	0.722	0.756	0.652	0.646	0.874

### Effects of CPB and CCB on service quality and satisfaction

To evaluate the hypothesized relationships (H1–H4), the structural model was analyzed by examining the direct effects of CPB and CCB on service quality dimensions and customer satisfaction. Table 8 presents the path coefficients, standard errors, t-statistics, and p-values for all tested relationships.

**Table 8:** Structural path coefficients for direct effects (CPB and CCB → Service Quality and Customer Satisfaction).

Relationship	Path Coefficient (β)	Standard Error (S.E.)	t-stat	p-value
CPB → CS	0.405	0.054	7.502	.000
CCB → CS	0.042	0.031	1.360	.174
CPB → PCQ	0.161	0.076	2.131	.033
CPB → CQ	0.213	0.053	4.053	.000
CPB → OQ	0.344	0.073	4.743	.000
CCB → PCQ	0.281	0.061	4.590	.000
CCB → CQ	0.407	0.048	8.400	.000
CCB → OQ	0.149	0.062	2.387	.017

The structural model in Table 8 reveals clear distinctions in how CPB and CCB influence CS and DSQ, composed of personal contact quality (PCQ), customization quality (CQ), and operational quality (OQ). Consistent with H1, CPB exerts a strong, positive, and statistically significant effect on customer satisfaction (β = 0.405, p < .001). This indicates that customers who actively engage, by providing precise delivery instructions, coordinating delivery schedules, and responding to notifications, directly enhance their service experience. Mechanistically, CPB improves informational accuracy, reduces uncertainty, and strengthens perceived control, which are critical pathways to satisfaction formation. These findings align with prior research in fast-growing e-commerce contexts, particularly in China, where active customer engagement in last-mile logistics has been shown to improve operational efficiency, reduce errors, and elevate perceived service quality [2–4].

CPB also significantly enhances all dimensions of DSQ, supporting H3, with the strongest effect observed in operational quality (β = 0.344), followed by customization (β = 0.213) and personal contact quality (β = 0.161). This pattern underscores the functional role of participation: customers directly contribute to more accurate, timely, and reliable service delivery while enabling tailored experiences. These results extend existing literature by demonstrating that participation behaviors are central not only to operational efficiency but also to overall satisfaction in high-demand urban logistics environments [9, 10].

In contrast, CCB does not exert a significant direct effect on customer satisfaction (β = 0.042, p = .174), offering partial support for H2. Voluntary behaviors, such as providing feedback, recommending services, or helping other customers, enhance relational aspects of service but do not immediately alter customers’ cognitive or affective evaluations. This finding contrasts with studies in hospitality and service sectors where CCB often directly improves satisfaction [12, 13], suggesting a contextual nuance in last-mile delivery: satisfaction is more sensitive to direct operational engagement than to voluntary, extra-role behaviors.

Nevertheless, CCB significantly improves all DSQ dimensions, supporting H4, particularly customization quality (β = 0.407) and personal contact quality (β = 0.281), with a moderate effect on operational quality (β = 0.149). These results indicate that voluntary behaviors cultivate a service environment characterized by personalized experiences, smooth interpersonal interactions, and adaptive responses. Such enhancements of DSQ indirectly support satisfaction by shaping the service context in which operational performance occurs. This aligns with prior research emphasizing the interactive nature of last-mile delivery and the role of co-creation behaviors in enriching relational and experiential outcomes [9, 10].

The findings highlight both similarities and distinctions from previous studies. Consistent with research on co-creation, CPB and CCB improve service quality and indirectly influence satisfaction [11]. However, unlike studies in other sectors where CCB directly enhances satisfaction, in last-mile delivery, operational engagement (CPB) drives immediate satisfaction, whereas citizenship behaviors contribute more subtly by improving personalization, relational quality, and adaptive service processes. This nuance is particularly relevant in the context of China’s rapidly growing e-commerce market and efficiency pressures make operational input a primary determinant of customer satisfaction [2, 3].

Overall, the structural results confirm that active customer participation is critical for immediate satisfaction, whereas citizenship behaviors play a vital role in shaping the service environment. Integrating these findings with prior studies highlights the necessity of distinguishing between functional and voluntary co-creation behaviors when designing digital service systems and strategies for last-mile delivery, particularly in high-demand urban e-commerce markets like China.

### Mediating effects of DSQ on the relationship between CVC and CS

Table 9 presents the mediating role of last-mile delivery service quality, comprising PCQ, CQ, and OQ, in the relationship between CPB, CCB, and CS. Both direct and indirect effects are reported, along with total effects, highlighting the pathways through which customer engagement translates into satisfaction.

**Table 9:** Mediating effects of last-mile delivery service quality on the relationship between customer value co-creation behaviors and customer satisfaction.

Relationship	Path Coefficient (β)	Standard Error (S.E.)	t-stat	p	Mediation Remarks
*Total Indirect Effects*					
CPB → PCQ → CS	0.023	0.014	1.669	.095	No effect
CPB → CQ → CS	0.025	0.011	2.283	.022	Partial Mediation
CPB → OQ → CS	0.099	0.022	4.425	.000	Partial Mediation
CCB → PCQ → CS	0.039	0.013	3.112	.002	Full Mediation
CCB → CQ → CS	0.047	0.016	3.000	.003	Full Mediation
CCB → OQ → CS	0.043	0.018	2.349	.019	Full Mediation
*Direct Effects*					
CPB → PCQ	0.161	0.076	2.131	.033	
CPB → CQ	0.213	0.053	4.053	.000	
CPB → OQ	0.344	0.073	4.743	.000	
CCB → PCQ	0.281	0.061	4.590	.000	
CCB → CQ	0.407	0.048	8.400	.000	
CCB → OQ	0.149	0.062	2.387	.017	
CPB → CS	0.405	0.054	7.502	.000	
CCB → CS	0.042	0.031	1.360	.174	
PCQ → CS	0.140	0.037	3.812	.000	
CQ → CS	0.116	0.037	3.170	.002	
OQ → CS	0.288	0.025	11.602	.000	
*Total Effects*					
CPB → CS	0.146	0.031	4.794	.000	
CCB → CS	0.130	0.028	4.563	.000	

The results indicate that DSQ serves as a crucial mechanism linking customer engagement to satisfaction. For CPB, the mediation analysis reveals that operational quality (OQ: β = 0.099, p < .001) and customization quality (CQ: β = 0.025, p < .05) partially mediate its effect on satisfaction, while personal contact quality (PCQ: β = 0.023, p = .095) does not significantly mediate. This suggests that the positive impact of participation behaviors, such as providing accurate delivery instructions, coordinating schedules, or assisting in the delivery process, is transmitted to satisfaction primarily through improvements in operational reliability and tailored services. In other words, CPB enhances customers’ evaluative outcomes indirectly by creating more accurate, reliable, and personalized delivery experiences. These findings confirm H5 and extend H3 by showing that CPB improves DSQ and, indirectly, satisfaction, but not all engagement dimensions yield direct benefits, aligning with the “dark side” perspective that co-creation is contingent on perceived convenience, effort, and value [43, 44, 47].

For CCB, all three DSQ dimensions fully mediate its effect on satisfaction (PCQ: β = 0.039, p < .01; CQ: β = 0.047, p < .01; OQ: β = 0.043, p < .05). Despite CCB having an insignificant direct effect on satisfaction (β = 0.042, p = .174), voluntary, extra-role behaviors, such as providing feedback, advocating for the service, or assisting other customers, enhance satisfaction indirectly by strengthening personalization, interpersonal interactions, and operational effectiveness. These results support H6 and complement H4, showing that citizenship behaviors rely entirely on improvements in service quality to influence satisfaction, again consistent with the contingent nature of co-creation.

Collectively, the total effects show that CPB (β = 0.146, p < .001) and CCB (β = 0.130, p < .001) significantly enhance satisfaction when accounting for the mediating role of DSQ. This underscores the complementary roles of functional and voluntary customer behaviors. CPB contributes mainly through operational and customization improvements, while CCB fosters relational, personalized, and process-oriented service enhancements. Overall, the findings highlight that while co-creation can enhance satisfaction, its effectiveness is contingent on the quality dimensions it influences, reflecting both the potential and limitations of customer engagement in last-mile delivery.

These findings align with prior literature on Service-Dominant Logic, which posits that value is co-created through the integration of resources and collaborative processes between customers and service providers [11]. Empirical studies in logistics, hospitality, and AI-enabled services indicate that customer engagement enhances satisfaction primarily through improved operational, relational, and personalized service qualities rather than through direct behavioral effects alone [62, 67–70].

In practice, these results suggest that logistics providers should actively facilitate both participation and citizenship behaviors. Encouraging CPB ensures operational accuracy and tailored service delivery, while fostering CCB strengthens relational interactions and personalization, collectively elevating last-mile service quality and, in turn, customer satisfaction. The mediating role of DSQ underscores the importance of investing in service processes and human–customer interaction mechanisms to fully capitalize on customer engagement as a driver of satisfaction in digitally enabled delivery environments.

### Mediating effects of digital accessibility

Table 10 presents the moderating effects of digital accessibility (DA) on the relationships between CPB, CCB, DSQ, and CS.

**Table 10:** Moderating effects of digital accessibility on the relationships between customer value co-creation behaviors, delivery service quality, and customer satisfaction.

Relationship	Path Coefficient (β)	Standard Error (S.E.)	t-stat	p-value
DA × CCB → CQ	0.087	0.039	2.252	.024
DA × CCB → CS	0.036	0.028	1.272	.203
DA × CCB → OQ	−0.017	0.053	0.325	.745
DA × CCB → PCQ	0.115	0.054	2.113	.035
DA × CPB → CQ	0.008	0.033	0.230	.818
DA × CPB → CS	0.061	0.034	1.780	.075
DA × CPB → OQ	0.150	0.050	2.986	.003
DA × CPB → PCQ	0.002	0.053	0.039	.969
DA → CQ	0.335	0.041	8.157	.000
DA → CS	0.214	0.029	7.299	.000
DA → OQ	0.353	0.051	6.859	.000
DA → PCQ	0.427	0.049	8.668	.000

The results indicate that digital accessibility significantly moderates several relationships, particularly in enhancing the effects of customer co-creation behaviors on service quality dimensions. For CCB, DA strengthens its impact on customization quality (CQ: β = 0.087, p < .05) and personal contact quality (PCQ: β = 0.115, p < .05), though its moderation on operational quality (OQ) and overall satisfaction (CS) is non-significant. This suggests that when digital platforms are easily accessible, voluntary customer behaviors—such as advocacy, feedback, or assisting others—more effectively translate into personalized services and better interpersonal interactions, reinforcing DSQ. These findings partially support H8 and H10.

For CPB, DA significantly moderates its effect on operational quality (OQ: β = 0.150, p < .05) but not on CQ, PCQ, or CS. This implies that the ease of using digital tools facilitates coordination, timely updates, and operational accuracy, amplifying the benefits of active, functional participation in the delivery process. However, the effect on satisfaction and personalization appears less pronounced, providing partial support for H7 and H9.

The direct effects of DA on all DSQ dimensions and CS are robust (CQ: β = 0.335; PCQ: β = 0.427; OQ: β = 0.353; CS: β = 0.214; all p < .001), highlighting the critical role of digital accessibility as an enabler of service quality and customer satisfaction independent of co-creation behaviors. These results are consistent with prior research suggesting that accessible digital platforms facilitate real-time tracking, interactive communication, and personalized engagement, thereby supporting operational and relational service outcomes [73, 74, 76].

At the theoretical level, these findings align with the Technology Acceptance Model [23], which posits that perceived ease of use and usefulness of digital tools increase both engagement and satisfaction. They also corroborate studies in last-mile logistics showing that digital platforms enable seamless customer participation in co-creation processes, enhancing operational reliability, personalization, and interpersonal interactions [23, 71, 75]. However, the non-significant moderation effects on satisfaction in some paths suggest that the influence of DA may be context-dependent, potentially moderated by cultural or operational factors, platform complexity, or information overload [65, 66].

In practice, these findings emphasize that logistics providers should prioritize user-friendly, accessible digital platforms to maximize the impact of both participation and citizenship behaviors on service quality. Operational coordination and personal contact quality, in particular, benefit from high digital accessibility, reinforcing the importance of integrating technology with behavioral engagement strategies to elevate last-mile service excellence.

### Model fitness

Table 11 presents the model fitness, showing both the explanatory (R²) and predictive (Q^2^) power of the structural model for last-mile delivery service quality dimensions and overall customer satisfaction.

**Table 11:** Model fitness: Explanatory and predictive power of last-mile delivery service quality and customer satisfaction.

Variables	R^2^	Model’s Explanatory Power	Q^2^	Model’s Predictive Capability
CQ	0.712	Moderate	0.613	Strong
OQ	0.468	Weak	0.374	Strong
PCQ	0.608	Moderate	0.490	Strong
CS	0.961	Substantial	0.756	Strong

The structural model demonstrates adequate fitness for explaining and predicting last-mile delivery service outcomes. The R^2^ values indicate that customer behaviors and digital accessibility explain substantial variance in customer satisfaction (R^2^ = 0.961), while DSQ dimensions vary in their explanatory power: customization quality (R^2^ = 0.712) and personal contact quality (R^2^ = 0.608) show moderate explanatory strength, whereas operational quality (R^2^ = 0.468) is relatively weaker. Despite the differences in R^2^, all dimensions exhibit strong predictive relevance (Q^2^ > 0.3), confirming the model’s ability to reliably forecast unseen data and underlying service quality effects on satisfaction. The model validates that customer behaviors shape satisfaction primarily through DSQ, supporting the mediating role of customization, operational, and personal contact quality as proposed in Service-Dominant Logic.

## Conclusions

The paper intended to clarify the process through which customer value co-creation takes place in last-mile delivery, an environment where minor efforts by customers have a higher impact than the companies want to believe. Two forms of conduct were examined here. One deals with involvement, such as providing correct delivery details, making delivery appointments, or reacting timely whenever there is an issue. The second is citizenship conduct, which is more extraverted and non-mandatory, such as providing assessment, being tolerant with delivery drivers, or promoting the services. The exploration also demanded a more complex question. Are these behaviors responsible for a rise in satisfaction on their own, or do they influence delivery service quality, and does anything get amplified or reduced by digital access? Results were tested with structural equation modeling with both moderation and mediation paths after given survey information of 426 online shoppers in urban China, which is above Cochran’s minimum suggestion comfortably.

Two key findings stand out as unambiguous. Customer participation behavior had a highly statistically significant and strong relationship to satisfaction. These results confirm H3–H6, demonstrating that co-creation behaviors enhance satisfaction primarily by improving operational reliability, personalized experiences, and relational interactions. On average, satisfaction grew as long as customers were engaged with the delivery process. This does not seem surprising given the average last-mile delivery experience. A customer who gives clear details about delivery, makes appointment times with accuracy, or responds quickly to a text message usually faces fewer problems and retarded delivery. It is the opposite case with citizenship behavior, which did not show a statistically significant direct influence on satisfaction. This does not mean it is insignificant. It shows that extra-role behavior, such as giving comments or showing tolerance at will, seems to be meaningful only to the degree that it influences service performance. In this regard, the patterns are consistent with previous studies that view participation and citizenship as two distinct but cognizable forms of engagement.

Moreover, digital access influenced specific patterns rather than increasing everything proportionately, supporting H7–H8. The positive influence of customer citizenship on personal contact quality and customization quality was magnified, along with the impact of participation behavior on operational quality as well. This can be compared to a well-structured delivery app that allows customers to text couriers with ease, change settings, or view parcel status in real-time. The tools themselves do not make customers happy independently, but they assist in making engagement transform into better services. In other words, digital access has only a minor moderating effect on direct links to satisfaction. It indicates that technology acts as an enabler rather than a shortcut. It makes co-creation increase services, and satisfaction is the indirect result.

The final takeaway is that satisfaction emerges through various subordinate chains that, instead of making individual contributions, come together. The main conclusions have implications in both theory and practice.

### Implications

Through the lens of theory, the paper solidifies Service-Dominant Logic, which claims that value emerges through customer actions and is then realized through quality of services, compatibility, and appropriateness. It also adds a technological component to co-creation research, which reveals that digital access clarifies the application of engagement instead of substituting it. Further, the research outcomes distinguish the different roles participation and citizenship behavior have as well. Whereas participation seems to have immediate effects on satisfaction, citizenship is mostly influenced by influencing service quality. For practitioners, the findings are clear. Firstly, logistics companies should demonstrate that the level of involvement should be straightforward and even enjoyable. Clear interfaces, flexible scheduling reference, and polite response to customer communication can improve satisfaction directly and service performance. Securing citizenship behavior, including sharing comments or interacting with delivery drivers, can improve personalization and relational quality even if the effect is low. The provision of straightforward and user-friendly digital platforms is a necessity, rather than a luxury, here. They are the way to make co-creation work, especially in terms of operational coordination and helping to provide customized service.

### Limitations

The study has limitations, though. The sample was urban consumers from Nanning, China, and it is unclear how representative or generalizable the findings are to other rural areas or countries. The cross-sectional design also prevents definitive conclusions about causality and longitudinal implications. Self-reported responses present the usual issues with recall and social desirability bias. Additionally, this study did not differentiate between delivery companies or residential community policies, which can influence service models, personalization, and access to last-mile delivery. This limitation has been acknowledged to highlight the need for future research to examine cross-company and cross-community variations to better capture contextual effects on co-creation behaviors and customer satisfaction. Finally, digital accessibility was operationalized as an overall baseline rather than defined for specific platform functions or degrees of technological proficiency. Future research might address these limitations by tracking customers longitudinally, expanding to other last-mile scenarios, and unpacking individual digital mechanisms in more detail. It would thereby add to insights about the lived experience of value co-creation in last-mile delivery.
