# Supplementary material for: Value co-creation and customer satisfaction in last-mile delivery services: The roles of digital accessibility and delivery service quality
Source: PLoS One. 2026 Oct 6;21(10):e0359308. doi: 10.1371/journal.pone.0359308 (PMC13641353; doi:10.1371/journal.pone.0359308)
Supplement: Supplementary file 1 [file pone.0359308.s002.docx]

**VALUE CO-CREATION AND CUSTOMER SATISFACTION IN LAST-MILE DELIVERY SERVICES: THE ROLES OF DIGITAL ACCESSIBILITY AND DELIVERY SERVICE QUALITY**

RESPONSE TO REVIEWER

| COMMENT | RESPONSE |
| --- | --- |
| Customers are inherently lazy and want more services from logistics companies, and the idea of co-creation through engagement would be unrealistic… It’s thus relevant to consider the dark side of co-creation/co-production. | In the revised manuscript, the potential “dark side” of co-creation we have incorporated in the literature review (lines 166 to 176; 189 to 192) and discussion sections (line 516-518; 524-526; and 531-534). Specifically, customer participation may increase perceived effort, shift responsibilities to consumers, and create resistance when engagement does not align with value-for-money expectations were acknowledged. The clarification about co-creation in last-mile delivery is context-dependent and may be constrained by customer convenience preferences (e.g., door-to-door vs. parcel lockers) were included. It was also highlighted that digital accessibility can mitigate perceived effort by reducing friction in engagement processes. |
| I want to see a chart to visualize the theoretical hypotheses. | A conceptual framework (Figure 1) illustrating all hypothesized relationships, including direct, mediating, and moderating effects was added. The diagram clearly presents CPB and CCB as antecedents, DSQ dimensions as mediators, digital accessibility as moderator, and customer satisfaction as the outcome variable. |
| In the survey, it is important to compare across communities and companies, given their differentiated policies and measures in logistics management. For instance, some communities prohibit logistics deliverymen to enter the apartments, not to mention door-to-door service. Shunfeng charges higher and give more personalized services than others (e.g., Shentong). Jingdong has its own logistics services, and they are well integrated with e-commerce. It would be biased to ignore these differences. | The comment regarding heterogeneity across logistics companies and residential community delivery policies is fully acknowledged. The current study did not collected data that would allow a formal comparison across delivery firms or community regulations. Therefore, the study was unable to control for or test these differences statistically. To resolve this, a statement in the Limitations section to acknowledge this limitation and highlight it as an avenue for future research was made, suggesting that subsequent studies could examine cross-company and cross-community variations to assess their influence on co-creation behaviors and customer satisfaction in last-mile delivery. |
| Readers might not be familiar with Nanning. Please briefly introduce this city and justify the case selection. | We have added a concise contextual description of Nanning in the methodology section, including its role as a rapidly developing urban center in southern China, its growing e-commerce penetration, and dense residential structures that intensify last-mile logistics challenges. We also clarified that Nanning provides a relevant urban setting characterized by high digital adoption and operational complexity, making it appropriate for examining co-creation in last-mile delivery. See line 281-285. |
| Please elaborate on how to address common method bias | To address this, the methodology section explicitly described both procedural and statistical remedies for common method bias (CMB). Procedurally, we ensured respondent anonymity, separated scale sections, and used established measurement items to reduce evaluation apprehension. Statistically, we conducted Harman’s single-factor test and assessed variance inflation factors (VIF) to detect potential bias. Results indicate that common method variance does not pose a significant threat to the findings. These details are now clearly reported in the revised manuscript.  To reduce concerns of common method bias, the study employed procedural and statistical remedies. Procedurally, survey items were adapted from validated instruments, carefully worded to reduce ambiguity, and responses were collected anonymously to minimize social desirability bias. Statistically, multicollinearity was assessed using the Variance Inflation Factor (VIF) (see Model Specification section, particularly the last paragraph). Table 4 shows all constructs exhibiting VIF values below the recommended threshold of 5 (Hair et al., 2022). Likewise indicating that common method bias is unlikely to significantly affect the results. |
