# Supplementary material for: Value co-creation and customer satisfaction in last-mile delivery services: The roles of digital accessibility and delivery service quality
Source: PLoS One. 2026 Oct 6;21(10):e0359308. doi: 10.1371/journal.pone.0359308 (PMC13641353; doi:10.1371/journal.pone.0359308)
Supplement: Supplementary file 2 [file pone.0359308.s003.docx]

**VALUE CO-CREATION AND CUSTOMER SATISFACTION IN LAST-MILE DELIVERY SERVICES: THE ROLES OF DIGITAL ACCESSIBILITY AND DELIVERY SERVICE QUALITY**

RESPONSE TO REVIEWER

| **Reviewer Comment** | **Response** | **Revision Made** |
| --- | --- | --- |
| **1. Weak Integration of the “Dark Side” of Co-Creation**. The manuscript briefly added statements about the “dark side” of co-creation, but these additions do not connect well with the overall narrative, theory, and findings. | We appreciate this insightful observation. We substantially revised the Introduction and Literature Review to integrate both the positive and negative perspectives of customer value co-creation. Rather than presenting the dark side as a standalone discussion, it is now positioned as a competing perspective that motivates the study. The revised manuscript explains that customer participation does not automatically generate positive outcomes and that its effectiveness depends on whether engagement contributes to meaningful improvements in service quality and customer satisfaction. We further connected this perspective to the interpretation of the mediation findings, demonstrating that co-creation behaviors influence satisfaction only when translated into improvements in specific service quality dimensions. | Revised the Introduction, Customer Value Co-Creation and Customer Satisfaction section, and the Discussion of Table 9 to integrate the contingent nature of co-creation and its implications for customer satisfaction. |
| **2. Too Many Literature Gaps Without Strong Justification**. The paper identifies six separate research gaps but does not sufficiently justify why these gaps matter theoretically or practically. | Thank you for this valuable suggestion. We streamlined the introduction by consolidating multiple gaps into three interconnected research gaps: (1) the distinct effects of customer participation and citizenship behaviors on satisfaction, (2) the mediating role of specific delivery service quality dimensions, and (3) the moderating role of digital accessibility. We also strengthened the theoretical and practical justification for addressing these gaps, particularly in the context of rapidly digitalizing last-mile delivery services. | Revised the Introduction to present a more focused and coherent research problem and strengthened the theoretical and practical rationale for the study. |
| **3. Some Claimed Literature Gaps Are Not Actually Addressed in the Study**. The manuscript claims to address operational improvements and organizational architectures, but these variables are not measured. | We agree with the reviewer. References to “operational improvements” and “organizational architectures” have been removed from the Introduction, Literature Review, Discussion, and Conclusions to ensure consistency between the stated research gaps and the variables included in the empirical model. | Removed unsupported claims throughout the manuscript and aligned the stated contributions with the actual constructs examined in the study. |
| **4. Delivery Service Quality (DSQ) Dimensions Are Oversimplified**. DSQ is measured as multiple subdimensions (PCQ, CQ, OQ), but the discussion treats DSQ as one overall construct. | We appreciate this important observation. The manuscript has been revised to consistently treat delivery service quality as a multidimensional construct composed of personal contact quality, customization quality, and operational quality. The Literature Review, mediation discussion, moderation discussion, and conclusions now explicitly discuss each dimension separately rather than treating DSQ as a single construct. | Revised the Literature Review, Tables 8–10 discussions, model fitness discussion, conclusions, and implications to explicitly distinguish PCQ, CQ, and OQ. |
| **5. Discussion Includes Variables Not Measured in the Model**. The paper discusses concepts such as trust, loyalty, fairness, convenience, effort–benefit balance, emotional response, organizational architectures, and operational improvements, but these are not measured in the study. | Thank you for highlighting this issue. We carefully reviewed the manuscript and removed or substantially reduced discussions of constructs not included in the empirical model. These concepts are now discussed only when referenced in prior literature and are no longer presented as findings or interpretations of the empirical results. | Revised the Literature Review, Results, Discussion, Conclusions, and Implications to ensure that interpretations remain grounded in the measured constructs. |
| **6. Interpretations Do Not Match the Data or Model**. Some conclusions discuss indirect effects, operational contexts, convenience, effort, and value even though these relationships or variables were not tested. | We appreciate this observation. The Results and Discussion sections were thoroughly revised to ensure that all interpretations are directly supported by the estimated structural model. Interpretations now focus exclusively on the tested relationships among customer participation behavior, customer citizenship behavior, digital accessibility, customer satisfaction, and the three delivery service quality dimensions. | Revised discussions for Tables 8, 9, and 10, as well as the Conclusions and Implications sections, to ensure alignment between empirical evidence and interpretation. |
| **7. Lack of Conceptual Consistency in Terminology**. Important constructs are labeled differently throughout the paper. | Thank you for this suggestion. We standardized terminology throughout the manuscript. Specifically, “operational quality” is now used consistently and references to operational efficiency, operational effectiveness, operational coordination, and similar terms were removed unless directly cited from prior literature. | Revised the manuscript to ensure consistent terminology across all sections. |
| **8. Weak Integration of Theories Into the Model**. The paper mentions Service-Dominant Logic, Dynamic Capabilities Theory, and Technology Acceptance Model, but these theories are only briefly discussed. | We appreciate this important observation. The theoretical framework was strengthened by explicitly linking Service-Dominant Logic to customer value co-creation behaviors, Dynamic Capabilities Theory to the role of digital accessibility in integrating customer inputs into service processes, and the Technology Acceptance Model to customer engagement through accessible digital platforms. These theoretical perspectives are now connected directly to the hypotheses and interpretation of findings. | Revised the Introduction, Literature Review, and moderation discussion to strengthen theoretical integration and improve alignment between theory and hypotheses. |
| **9. Hypotheses Lack Clear Theoretical Support**. Hypotheses H1–H4 are not strongly connected to any theoretical foundation. | Thank you for this constructive comment. We strengthened the theoretical justification for H1–H4 by grounding the hypotheses in Service-Dominant Logic and customer value co-creation literature. The revised discussion explains how participation and citizenship behaviors contribute to value creation through customer-provider interactions and how these processes are expected to influence service quality and satisfaction. | Revised the Customer Value Co-Creation sections to provide stronger theoretical support for H1–H4. |
| **10. Narrative and Storyline Need Stronger Coherence**. The manuscript contains many concepts and theories that are not woven together into a coherent narrative. | We appreciate this observation. The manuscript was reorganized to establish a clearer narrative flow from the challenges of customer satisfaction in last-mile delivery, to customer value co-creation, delivery service quality, digital accessibility, and customer satisfaction. The revised manuscript now presents a more coherent explanation of how behavioral and technological factors jointly influence service outcomes. | Revised the Introduction, Literature Review, Discussion, and Conclusions to improve conceptual coherence and logical flow. |
| **11. Contribution to Theory and Practice Needs Stronger Positioning**. The manuscript does not clearly articulate its unique contribution. | Thank you for this valuable suggestion. We strengthened the positioning of the study’s contributions by emphasizing three key contributions: (1) distinguishing the effects of participation and citizenship behaviors, (2) examining the mediating roles of personal contact quality, customization quality, and operational quality separately, and (3) investigating the selective moderating role of digital accessibility. Practical implications for logistics providers were also clarified. | Revised the Introduction, Conclusions, and Implications sections to better articulate theoretical and practical contributions. |
| **12. Overall Writing and Readability Need Improvement**. | We appreciate this recommendation. The manuscript underwent extensive editing to improve clarity, consistency, readability, and conceptual organization. Redundant discussions were removed, terminology was standardized, and several sections were reorganized to improve logical flow. | Revised the manuscript throughout to improve readability, conceptual clarity, and overall presentation. |
