# Supplementary material for: Value co-creation and customer satisfaction in last-mile delivery services: The roles of digital accessibility and delivery service quality
Source: PLoS One. 2026 Oct 6;21(10):e0359308. doi: 10.1371/journal.pone.0359308 (PMC13641353; doi:10.1371/journal.pone.0359308)
Supplement: Supplementary file 3 [file pone.0359308.s004.docx]

**RESPONSE TO REVIEWER #3**

**Response to Reviewers and Summary of Revisions**

We sincerely thank Reviewer #3 for the careful and constructive assessment of the revised manuscript. The comments have helped us further improve the manuscript's conceptual clarity, methodological transparency, terminological consistency, and alignment between the conceptual framework, hypotheses, analyses, results, and conclusions. We have revised the manuscript accordingly while retaining the original study design, dataset, and analytical approach. The responses below identify the revisions made and the corresponding sections of the manuscript.

**General Revision Note.** Throughout the revised manuscript, Delivery Service Quality (DSQ) is treated as a conceptual multidimensional construct comprising Personal Contact Quality (PCQ), Customization Quality (CQ), and Operational Quality (OQ). The empirical analyses examine PCQ, CQ, and OQ separately. The hypothesis numbering and discussion have therefore been aligned with the dimensional analyses reported in Tables 8–10.

| **Reviewer Comment** | **Response** | **Revision Made** | **Location in Revised Manuscript** |
| --- | --- | --- | --- |
| 1. Introduction and Literature Review  The research gap and the study's significance are insufficiently articulated. The authors are encouraged to include a summary at the end of the Introduction or Literature Review that clearly highlights the research gap, the study's significance, and its contribution to the existing literature. | Thank you for this important observation. We agreed that the previous presentation identified several gaps without sufficiently explaining how they formed a coherent research problem. We therefore consolidated the gaps into three interconnected gaps that directly correspond to the empirical model. | The Introduction was revised to present three connected gaps: (1) the distinct effects of CPB and CCB on customer satisfaction in last-mile delivery; (2) the separate mediating roles of PCQ, CQ, and OQ; and (3) the selective moderating role of DA. We also added an explicit explanation of why these gaps matter theoretically and practically, emphasizing that last-mile delivery increasingly depends on interactions among customers, providers, and digital platforms. The concluding paragraphs of the Introduction now explicitly state the study's theoretical and practical contributions. | Introduction – research gap, study significance, and contribution paragraphs |
| 2. Conceptual Framework and Hypotheses  The presentation of the hypotheses and conceptual framework could be improved. The terminology used to describe the same construct is not consistent. H3 and H4 refer to last-mile delivery service quality, whereas H7–H10 use delivery service quality. The framework should use consistent terminology, include H1–H10 labels, distinguish CPB and CCB paths, and present abbreviations consistently. | We appreciate this observation and have revised the conceptual presentation accordingly. The formal construct terminology is now standardized as Delivery Service Quality (DSQ), with PCQ, CQ, and OQ identified as its three dimensions. The phrase “last-mile delivery service quality” is retained only as contextual language and is no longer used as an alternative construct label. | The conceptual framework has been revised to: (a) use DSQ consistently; (b) show PCQ, CQ, and OQ as the three DSQ dimensions; (c) distinguish CPB and CCB paths separately; (d) display hypothesis labels on the corresponding paths; and (e) present abbreviations consistently, including CCB and DA. The hypothesis structure was also revised to H3a–H3c, H4a–H4c, H5a–H5c, H6a–H6c, H7a–H7c, and H8a–H8c to match the dimensional analyses. | Conceptual Framework and Hypotheses; revised research model and hypothesis development |
| 3. Methodology and Participant Characteristics  The demographic table should report frequencies and totals consistently. The manuscript also states that demographic information includes region, but region is not reported in the table or Results section. Please clarify whether regional information was collected. | Thank you. We reviewed the demographic reporting and revised the presentation to ensure that the sample description corresponds exactly to the information actually collected and reported. We have avoided retaining a demographic category that is not supported by the reported results. | The demographic table was revised to report frequencies and percentages consistently across the reported demographic variables, with the total sample of 426 respondents clearly indicated. The manuscript's description of demographic information was also revised so that it does not imply the collection/reporting of regional information unless such information is actually presented in the results. | Methodology – Participant Characteristics / Table 2 |
| 4. Ethical Approval  The manuscript states that ethical approval was obtained from De La Salle University. If available, the authors are encouraged to report the approval/reference number. | We agree that reporting the institutional ethics committee more specifically improves transparency. The ethics statement has therefore been clarified. | The revised Ethics statement identifies the approving body as the De La Salle University–Dasmariñas Research Ethics Committee. The statement also clarifies that approval was obtained before data collection, informed consent was obtained electronically through the online survey, all participants were adults aged 18 years or older, no minors were involved, and no waiver of consent was granted. An approval/reference number is not reported because no such number is available in the study documentation currently being reported. | Ethical Approval and Participant Consent |
| 5. Results Presentation – Descriptive Results  Table 3 currently reports item-level descriptive statistics, making it difficult to obtain an overall understanding of each construct. The authors may consider presenting descriptive statistics at the construct level or organizing the results by construct. | We appreciate this recommendation. We revised the presentation so that readers can more readily interpret the descriptive characteristics of the measured constructs without losing the underlying item-level information where it is needed for measurement assessment. | The descriptive-results presentation was reorganized to improve construct-level readability. The narrative now emphasizes the overall descriptive characteristics of the constructs, while item-level information is retained where appropriate for measurement-model reporting. | Results – Descriptive Statistics / Table 3 |
| 6. Measurement Model Reporting  The measurement model is presented in the tables, including factor loadings, Cronbach's alpha, composite reliability, AVE, and discriminant validity. The accompanying text provides only a general description. Please briefly summarize key findings and support evaluation criteria with methodological references such as Hair et al. or Fornell and Larcker. | We agree. The measurement-model discussion was expanded to interpret the reported values rather than merely directing readers to the tables. | The revised measurement-model section now summarizes the observed ranges and findings for factor loadings, Cronbach's alpha, composite reliability, and AVE and explicitly relates them to established criteria. Discriminant validity is also discussed using the appropriate assessment criteria and supported by methodological references, including Hair et al. and Fornell and Larcker where applicable. | Measurement Model – Tables 4–7 and accompanying discussion |
| 7. Table Presentation  Tables 6 and 7 should clearly define all abbreviations used, preferably in table notes or captions, and provide full construct names for readability. | We agree and have standardized the table notes so that readers can interpret the tables without repeatedly referring to earlier sections. | Table notes/captions were revised to define the construct abbreviations consistently. The relevant notes now identify CPB, CCB, DA, PCQ, CQ, OQ, and CS using their full construct names. This convention is applied consistently across the measurement and structural-model tables. | Tables 6 and 7 – revised notes/captions |
| 8. Hypothesis Development and Results Reporting  H3 and H4 are proposed at the DSQ construct level, but the analyses are reported at the dimensional level (PCQ, CQ, OQ). The same issue occurs for H5 and H6. The authors should either analyze the aggregate construct or reformulate the hypotheses at the dimensional level. | We agree with the reviewer that the original wording created a mismatch between the hypotheses and the empirical analyses. Because the reported PLS-SEM model estimates PCQ, CQ, and OQ separately, we revised the hypotheses rather than introducing an unreported aggregate DSQ analysis. | The hypotheses were reformulated as follows: H3a–H3c test CPB → PCQ/CQ/OQ; H4a–H4c test CCB → PCQ/CQ/OQ; H5a–H5c test CPB → PCQ/CQ/OQ → CS; and H6a–H6c test CCB → PCQ/CQ/OQ → CS. The moderation hypotheses were similarly revised to H7a–H7c and H8a–H8c for the three DSQ dimensions, while H9 and H10 retain the moderation paths to CS. The Model Specification section now provides equations corresponding to these paths. Tables 8–10 and the Results, Discussion, and Conclusions have been revised to use the same dimensional structure. | Model Specification; Hypothesis Development; Tables 8–10; Results and Discussion; Conclusions |
| 9. Consistency in Mediation/Moderation Terminology  The manuscript refers to digital accessibility as having “mediating effects,” although it is proposed and analyzed as a moderator. Please revise the terminology consistently. | Thank you for identifying this terminology error. We agree that digital accessibility is a moderator in the conceptual and statistical model and should not be described as a mediator. | All relevant sections were reviewed and revised. Digital accessibility is now consistently described as a moderating variable, and the results are reported as “moderating effects” or “interaction effects.” The manuscript no longer refers to DA as a mediator. The moderation discussion also now explicitly distinguishes the significant interaction effects from the non-significant moderation effects on customer satisfaction. | Conceptual Framework; Model Specification; Table 10 Discussion; Conclusions and Implications |

**Additional cross-manuscript revisions**

**Terminology:** The manuscript consistently uses Delivery Service Quality (DSQ) as the conceptual multidimensional construct and PCQ, CQ, and OQ as its separately analyzed dimensions.

**Direct effects:** Table 8 and its discussion now report H1–H4 at the level actually estimated, with H3a–H3c and H4a–H4c explicitly identified.

**Mediation:** Table 9 is now focused on specific indirect effects. The discussion reports H5a–H5c and H6a–H6c separately rather than treating DSQ as a single mediator.

**Moderation:** Table 10 and its discussion report H7a–H7c, H8a–H8c, H9, and H10 separately. DA is consistently treated as a moderator.

**Model fitness:** Table 11 is interpreted at the level of PCQ, CQ, OQ, and CS rather than implying a single aggregate DSQ R² or Q².

**Conclusions and implications:** The conclusions and implications were revised to reflect the exact supported and unsupported hypotheses and the dimensional mediation/moderation results.

**Dark-side perspective:** The Introduction and interpretation now frame co-creation as potentially beneficial but contingent on whether customer engagement is translated into service-quality outcomes, avoiding claims that co-creation is universally beneficial.

**Scope of claims:** Claims concerning unmeasured variables such as organizational architectures, trust, loyalty, fairness, convenience, effort-benefit balance, and independent operational improvements have been removed or reframed as contextual considerations rather than tested variables.

We again thank Reviewer #3 for the constructive recommendations. The revisions have been made with particular attention to ensuring that the conceptual framework, hypotheses, statistical model, tables, interpretation, and conclusions describe the same empirical relationships. We believe these changes substantially improve the manuscript's conceptual coherence, methodological transparency, and readability while preserving the study's original design and contribution.
