# Supplementary material for: Value co-creation and customer satisfaction in last-mile delivery services: The roles of digital accessibility and delivery service quality
Source: PLoS One. 2026 Oct 6;21(10):e0359308. doi: 10.1371/journal.pone.0359308 (PMC13641353; doi:10.1371/journal.pone.0359308)
Supplement: Peer review history

# Peer review: Decision Letter 0

**DOI:** 10.1371/journal.pone.0359308.r001

**Authors:** Steve Zimmerman

25 Feb 2026

Dear Dr. Manansala,

Thank you for submitting your manuscript to PLOS ONE. After careful consideration, we feel that it has merit but does not fully meet PLOS ONE’s publication criteria as it currently stands. Therefore, we invite you to submit a revised version of the manuscript that addresses the points raised during the review process.

In addition to addressing the reviewers comments (below), please can you also provide a copy of your questionnaire?

Please submit your revised manuscript by Apr 10 2026 11:59PM. If you will need more time than this to complete your revisions, please reply to this message or contact the journal office at ?>plosone@plos.org. When you're ready to submit your revision, log on to https://www.editorialmanager.com/pone/ and select the 'Submissions Needing Revision' folder to locate your manuscript file.

- A letter that responds to each point raised by the academic editor and reviewer(s). You should upload this letter as a separate file labeled 'Response to Reviewers'.
- A marked-up copy of your manuscript that highlights changes made to the original version. You should upload this as a separate file labeled 'Revised Manuscript with Track Changes'.
- An unmarked version of your revised paper without tracked changes. You should upload this as a separate file labeled 'Manuscript'.

If applicable, we recommend that you deposit your laboratory protocols in protocols.io to enhance the reproducibility of your results. Protocols.io assigns your protocol its own identifier (DOI) so that it can be cited independently in the future. For instructions see: https://journals.plos.org/plosone/s/submission-guidelines#loc-laboratory-protocols. Additionally, PLOS ONE offers an option for publishing peer-reviewed Lab Protocol articles, which describe protocols hosted on protocols.io. Read more information on sharing protocols at https://plos.org/protocols?utm_medium=editorial-email&utm_source=authorletters&utm_campaign=protocols.

We look forward to receiving your revised manuscript.

Kind regards,

Steve Zimmerman, PhD

Senior Editor, PLOS One

Journal Requirements:

When submitting your revision, we need you to address these additional requirements.

1. Please ensure that your manuscript meets PLOS ONE's style requirements, including those for file naming. The PLOS ONE style templates can be found at

https://journals.plos.org/plosone/s/file?id=wjVg/PLOSOne_formatting_sample_main_body.pdfand https://journals.plos.org/plosone/s/file?id=ba62/PLOSOne_formatting_sample_title_authors_affiliations.pdf

2. We note that the grant information you provided in the ‘Funding Information’ and ‘Financial Disclosure’ sections do not match.

When you resubmit, please ensure that you provide the correct grant numbers for the awards you received for your study in the ‘Funding Information’ section.

3. Thank you for stating the following financial disclosure:

[The 2024 Guangxi Vocational Education Teaching Reform Research Project (Grant No. GXGZJG2024B001)

The 2024 Guangxi Higher Education Undergraduate Teaching Reform Project (Grant No. 2024JGB351].

Please state what role the funders took in the study.  If the funders had no role, please state: "The funders had no role in study design, data collection and analysis, decision to publish, or preparation of the manuscript."

If this statement is not correct you must amend it as needed.

Please include this amended Role of Funder statement in your cover letter; we will change the online submission form on your behalf.

4. In the online submission form, you indicated that [The data underlying the results presented in the study are available upon request to the corresponding author.].

All PLOS journals now require all data underlying the findings described in their manuscript to be freely available to other researchers, either 1. In a public repository, 2. Within the manuscript itself, or 3. Uploaded as supplementary information.

This policy applies to all data except where public deposition would breach compliance with the protocol approved by your research ethics board. If your data cannot be made publicly available for ethical or legal reasons (e.g., public availability would compromise patient privacy), please explain your reasons on resubmission and your exemption request will be escalated for approval.

5. When completing the data availability statement of the submission form, you indicated that you will make your data available on acceptance. We strongly recommend all authors decide on a data sharing plan before acceptance, as the process can be lengthy and hold up publication timelines. Please note that, though access restrictions are acceptable now, your entire data will need to be made freely accessible if your manuscript is accepted for publication. This policy applies to all data except where public deposition would breach compliance with the protocol approved by your research ethics board. If you are unable to adhere to our open data policy, please kindly revise your statement to explain your reasoning and we will seek the editor's input on an exemption. Please be assured that, once you have provided your new statement, the assessment of your exemption will not hold up the peer review process.

If the reviewer comments include a recommendation to cite specific previously published works, please review and evaluate these publications to determine whether they are relevant and should be cited. There is no requirement to cite these works unless the editor has indicated otherwise.

Please review your reference list to ensure that it is complete and correct. If you have cited papers that have been retracted, please include the rationale for doing so in the manuscript text, or remove these references and replace them with relevant current references. Any changes to the reference list should be mentioned in the rebuttal letter that accompanies your revised manuscript. If you need to cite a retracted article, indicate the article’s retracted status in the References list and also include a citation and full reference for the retraction notice.

[Note: HTML markup is below. Please do not edit.]

Reviewers' comments:

Reviewer's Responses to Questions

**Comments to the Author**

1. Is the manuscript technically sound, and do the data support the conclusions?

Reviewer #1: Yes

**********

2. Has the statistical analysis been performed appropriately and rigorously?

Reviewer #1: Yes

**********

3. Have the authors made all data underlying the findings in their manuscript fully available?

The PLOS Data policy

Reviewer #1: No

**********

4. Is the manuscript presented in an intelligible fashion and written in standard English?

Reviewer #1: Yes

**********

Reviewer #1: In this study, the authors used survey data to explore how customer engagement helps to enhance logistics service satisfaction. The findings are theoretically justified and empirically corroborated. Here I give a couple of suggestions for them to improve it.

1. Customers are inherently lazy and want more services from logistics companies, and the idea of co-creation through engagement would be unrealistic. For instance, customers want door-to-door service instead of parcel lockers. As long as it’s about value for money, customers would not lose the bargains with logistics companies. It’s thus relevant to consider the dark side of co-creation/co-production.

2. I want to see a chart to visualize the theoretical hypotheses.

3. In the survey, it is important to compare across communities and companies, given their differentiated policies and measures in logistics management. For instance, some communities prohibit logistics deliverymen to enter the apartments, not to mention door-to-door service. Shunfeng charges higher and give more personalized services than others (e.g., Shentong). Jingdong has its own logistics services, and they are well integrated with e-commerce. It would be biased to ignore these differences.

4. The readers might not be familiar with Nanning, and it merits to briefly introduce this city and justify this case selection.

5. Please elaborate on how to address the common method bias in survey studies.

**********

what does this mean?). If published, this will include your full peer review and any attached files.

If you choose “no”, your identity will remain anonymous but your review may still be made public.

**Do you want your identity to be public for this peer review?** For information about this choice, including consent withdrawal, please see our Privacy Policy

Reviewer #1: **Yes:**  Liang Ma

**********

[NOTE: If reviewer comments were submitted as an attachment file, they will be attached to this email and accessible via the submission site. Please log into your account, locate the manuscript record, and check for the action link "View Attachments". If this link does not appear, there are no attachment files.]

To ensure your figures meet our technical requirements, please review our figure guidelines: https://journals.plos.org/plosone/s/figures

You may also use PLOS’s free figure tool, NAAS, to help you prepare publication quality figures: https://journals.plos.org/plosone/s/figures#loc-tools-for-figure-preparation.

NAAS will assess whether your figures meet our technical requirements by comparing each figure against our figure specifications.

# Author comment: Author response to Decision Letter 1

**DOI:** 10.1371/journal.pone.0359308.r002

3 Mar 2026

Dear Editor and Reviewer

We sincerely thank you and the reviewers for the constructive and insightful feedback on our manuscript. We greatly appreciate the time and expertise invested in the review process. We have carefully revised the manuscript in response to all comments and have provided a detailed, point-by-point explanation in the accompanying Response to Reviewers document.

The major revisions include: (1) incorporating a balanced discussion on the potential “dark side” and contingent nature of customer co-creation in both the literature review and discussion sections; (2) adding a conceptual framework figure to clearly visualize the hypothesized relationships; (3) acknowledging cross-company and cross-community heterogeneity as a limitation and future research direction; (4) expanding the description and justification of Nanning as the study context; and (5) elaborating on procedural and statistical remedies for common method bias, including reporting VIF and Harman Single Factor results.

This research was supported by (1) The 2024 Guangxi Vocational Education Teaching Reform Research Project (Grant No. GXGZJG2024B001) and (2) The 2024 Guangxi Higher Education Undergraduate Teaching Reform Project (Grant No. 2024JGB351). The funders had a role in the study design, data collection and analysis, decision to publish, and preparation of the manuscript.

Furthermore, all authors have approved the revised manuscript and agree to its submission. The sharing of data associated with this study has been reviewed and approved by all authors. The manuscript is original, has not been published elsewhere, and is not under consideration by any other journal.

Thank you for your consideration. We look forward to your response.

Sincerely,

Attachment Submitted filename: RESPONSE TO REVIEWER.docx

# Peer review: Decision Letter 1

**DOI:** 10.1371/journal.pone.0359308.r003

**Authors:** Cheong Kim

26 May 2026

Dear Dr. Manansala,

Thank you for submitting your manuscript to PLOS ONE. After careful consideration, we feel that it has merit but does not fully meet PLOS ONE’s publication criteria as it currently stands. Therefore, we invite you to submit a revised version of the manuscript that addresses the points raised during the review process.

Please submit your revised manuscript by Jul 10 2026 11:59PM. If you will need more time than this to complete your revisions, please reply to this message or contact the journal office at plosone@plos.org. When you're ready to submit your revision, log on to https://www.editorialmanager.com/pone/ and select the 'Submissions Needing Revision' folder to locate your manuscript file.

- A letter that responds to each point raised by the academic editor and reviewer(s). You should upload this letter as a separate file labeled 'Response to Reviewers'.
- A marked-up copy of your manuscript that highlights changes made to the original version. You should upload this as a separate file labeled 'Revised Manuscript with Track Changes'.
- An unmarked version of your revised paper without tracked changes. You should upload this as a separate file labeled 'Manuscript'.

If you would like to make changes to your financial disclosure, please include your updated statement in your cover letter. Guidelines for resubmitting your figure files are available below the reviewer comments at the end of this letter.

If applicable, we recommend that you deposit your laboratory protocols in protocols.io to enhance the reproducibility of your results. Protocols.io assigns your protocol its own identifier (DOI) so that it can be cited independently in the future. For instructions see: https://journals.plos.org/plosone/s/submission-guidelines#loc-laboratory-protocols. Additionally, PLOS ONE offers an option for publishing peer-reviewed Lab Protocol articles, which describe protocols hosted on protocols.io. Read more information on sharing protocols at https://plos.org/protocols?utm_medium=editorial-email&utm_source=authorletters&utm_campaign=protocols.

As the corresponding author, your ORCID iD is verified in the submission system and will appear in the published article. PLOS supports the use of ORCID, and we encourage all coauthors to register for an ORCID iD and use it as well. Please encourage your coauthors to verify their ORCID iD within the submission system before final acceptance, as unverified ORCID iDs will not appear in the published article. *Only* the individual author can complete the verification step; PLOS staff *cannot* verify ORCID iDs on behalf of authors.

We look forward to receiving your revised manuscript.

Kind regards,

Cheong Kim

Academic Editor

PLOS One

Journal Requirements:

If the reviewer comments include a recommendation to cite specific previously published works, please review and evaluate these publications to determine whether they are relevant and should be cited. There is no requirement to cite these works unless the editor has indicated otherwise.

Additional Editor Comments :

Please carefully address the comments raised by Reviewer #2 in your revised manuscript. We kindly ask you to provide a detailed point-by-point response explaining how each issue has been addressed.

[Note: HTML markup is below. Please do not edit.]

Reviewers' comments:

Reviewer's Responses to Questions

**Comments to the Author**

Reviewer #1: All comments have been addressed

Reviewer #2: (No Response)

**********

2. Is the manuscript technically sound, and do the data support the conclusions?

Reviewer #1: Yes

Reviewer #2: Partly

**********

3. Has the statistical analysis been performed appropriately and rigorously?

Reviewer #1: Yes

Reviewer #2: Yes

**********

4. Have the authors made all data underlying the findings in their manuscript fully available?

The PLOS Data policy

Reviewer #1: No

Reviewer #2: Yes

**********

5. Is the manuscript presented in an intelligible fashion and written in standard English?

Reviewer #1: Yes

Reviewer #2: No

**********

Reviewer #1: Thanks for the responses. I think the authors have successfully respond to my comments and I'm happy with the revisions.

Reviewer #2: Thank you for the opportunity to review your work "Value Co-Creation and Customer Satisfaction in Last-Mile Delivery Services: The roles of digital accessibility and delivery service quality" for PLOS One. I was not a reviewer for the original manuscript and so read the revision first, without looking at the original review comments. Following my initial reading, I went through the reviewer comments to assess the authors' response. I won't go through each individual comment, but will say that the authors have largely responded appropriately to the comments presented.

The response to the reviewer's comment "Customers are inherently lazy and want more service from logistics companies, and the idea of co-creation through engagement would be unrealistic...It's thus relevant to consider the dark side of co-creation/co-production" is to add a couple of statements about the dark side into the manuscript. While this moves the paper in the right direction, reading the paper without the knowledge of this reviewer comment made it difficult to fully understand why the authors discussed the dark side of co-creation. The statements did not connect with the overall story of the paper and the data. Spending some more time trying to work through a coherent narrative that includes the dark side may help the paper's readability and contribution significantly.

When reading through a revision, I try to focus on the comments and changes that arise from the initial review. As this is my first read through the paper, I feel it necessary to add comments beyond those concerns raised in the initial review.

The authors present an interesting area, with a recent push toward "consumer logistics" or value co-creation as an important topic for logistics research, especially in last-mile delivery. At the same time, issues regarding the writing and use of theory in the paper should be addressed.

1. The authors mention six separate gaps in prior literature:

Operational efficiency does not thoroughly account for variations in customer satisfaction (line 33)

Little prior research addresses how CVC behaviors drive each dimension and mediate the link between co-creation and satisfaction (line 57)

It remains underexamined whether operational improvements function in last-mile delivery, constituting an obvious gap in the literature (line 60)

It remains questionable whether digital accessibility strengthens or inhibits the impact of CVC on delivery service quality and satisfaction (line 73)

While prior literature highlights customer engagement, it has yet to explore how these organizational architectures drive the impacts of CVC behaviors on delivery service quality and satisfaction (line 88)

Empirical evidence integrating participation and citizenship behaviors, multi-faceted service quality, and digital accessibility as an integrated approach remains scarce (line 118)

1a. While these may constitute gaps in the literature, the authors should provide some justification for the reason to examine these gaps. Explaining how these different concepts fit together with theory, then arguing that the theory should be tested would provide support. Highlighting practitioner issues, either through informal conversations, structured interviews, or popular press would also highlight the importance of the research.

1b. The data and analysis employed in the paper respond to some of the stated gaps, but the authors do not examine operational improvements (line 60) or organizational architectures (line 88), leaving two gaps unanswered. If the authors have data to help fill in these gaps, they should incorporate that into the analysis. Otherwise, it may be better to remove these statements from the paper.

2. The authors measure delivery service quality (DSQ) as three separate constructs (PCQ, CQ, and OQ) (first mentioned on line 50), but then treat DSQ as a single construct in the discussion. The authors, at one point, even point out that "not all engagement dimensions yield direct benefits" (line 481) when explaining results for the connection between CPB and DSQ. The fact that the authors measured multiple dimensions of quality and that some of the sub-dimensions exhibited different influences could provide a greater contribution. Instead of treating them as a single construct, highlighting and trying to explain these differences would improve the paper's contribution.

3. Several statements made in the paper don't align with the data, analysis, and results. For example:

3a. The authors mentioned several constructs that aren't measured in the model. Bringing out these different constructs without measuring them can detract from the overall story and contribution. The authors mention "organizational architectures" (line 88), "operational improvements" (line 60), "digital service design" (line 69), "perceived fairness, convenience, and effort-benefit balance" (line 135), "trust" (line 159), "loyalty" (line 159), "environments with scarce options" (line 163), "emotional response" (line 166), "meeting of expectations" as distinct from performance or satisfaction (line 166). None of these concepts are measured or aligned with the theories utilized in the paper.

3b. "Such enhancements of DSQ indirectly support satisfaction by shaping the service context in which operational performance occurs" (line 447). The authors test a direct relationship between DSQ and satisfaction with moderating effects from digital accessibility. The statement arguing for an indirect connection defined through service context and operational performance do not emerge from the data or the model. The authors should adjust their description of the moderated relationship in terms of digital accessibility and DSQ without the additional discussion of service context and explain the how changes in DA influence the relationship between DSQ and satisfaction.

3c. The authors state that "These findings confirm H5 and H3 by showing that CPB improves DSQ and indirectly, satisfaction, but not all engagement dimensions yield direct benefits, aligning with the "dark side" perspective that co-creation is contingent on perceived convenience, effort, and value" (line 481). The authors use the connection between CPB, one form of engagement, the three sub-dimensions of DSQ, and satisfaction. They then make a statement about convenience, effort, and value, which are not measured in the model.

4. The authors should seek conceptual consistency throughout the paper. Several of their important constructs are labelled differently throughout the manuscript. For instance, operational quality is labeled "reliability" "operational efficiency" "operational effectiveness" and "operational coordination" at different points in the paper. While this removes redundancy, it makes it more difficult to connect the narrative to the model. Ensuring consistent use of construct labels will aid readability and more clearly direct the reader to the main contributions.

5. The authors list three separate theories as guiding their work, but these are mentioned briefly and associated with a couple of hypotheses instead of helping to outline the connections between constructs or explain the mechanisms associated with the relationships. The authors mention service dominant logic in the introduction (line 100 ) and use the theory to support hypotheses 5 and 6 (line 220). Similarly the authors reference dynamic capabilities in the introduction (line 100), but don't connect dynamic capabilities to the overall model or the hypotheses. Finally, the authors introduce the technology acceptance model to support hypotheses 7 through 10 (line 228). This leaves hypotheses 1 through 4 without theoretical support and dynamic capabilities disconnected from the model. Explaining the need for multiple theories, providing some discussion of the different theories, and using those theories to organize and streamline the paper's narrative would greatly strengthen the authors' contribution.

In conclusion, the authors examine an important area in value co-creation in last-mile delivery, but the manuscript requires significant work to more clearly tell their story and outline their contributions to theory and practice. I hope the authors view my comments as constructive and thank the authors again for the opportunity to review their work!

**********

what does this mean?). If published, this will include your full peer review and any attached files.

If you choose “no”, your identity will remain anonymous but your review may still be made public.

**Do you want your identity to be public for this peer review?** For information about this choice, including consent withdrawal, please see our Privacy Policy

Reviewer #1: **Yes:**  Liang Ma

Reviewer #2: No

**********

[NOTE: If reviewer comments were submitted as an attachment file, they will be attached to this email and accessible via the submission site. Please log into your account, locate the manuscript record, and check for the action link "View Attachments". If this link does not appear, there are no attachment files.]

To ensure your figures meet our technical requirements, please review our figure guidelines: https://journals.plos.org/plosone/s/figures

You may also use PLOS’s free figure tool, NAAS, to help you prepare publication quality figures: https://journals.plos.org/plosone/s/figures#loc-tools-for-figure-preparation.

NAAS will assess whether your figures meet our technical requirements by comparing each figure against our figure specifications.

# Author comment: Author response to Decision Letter 2

**DOI:** 10.1371/journal.pone.0359308.r004

8 Jun 2026

Greetings.

We would like to thank you and the reviewers for the careful evaluation of our manuscript and for the constructive comments and suggestions. We have thoroughly revised the manuscript and believe that the changes have substantially improved its theoretical grounding, conceptual coherence, methodological transparency, and overall readability.

In response to the reviewers’ recommendations, we made several important revisions. First, we strengthened the theoretical narrative by integrating the positive and negative perspectives of customer value co-creation, including a more explicit discussion of the contingent or “dark side” nature of co-creation within the context of last-mile delivery services. Second, we streamlined and consolidated the literature gaps to provide a clearer justification of the study’s theoretical and practical significance. Third, we removed references to concepts not measured in the empirical model and aligned all discussions, interpretations, and conclusions with the actual constructs examined in the study.

We also enhanced the conceptual framework by more explicitly linking Service-Dominant Logic, Dynamic Capabilities Theory, and the Technology Acceptance Model to the proposed hypotheses and empirical findings. The discussions of the results were revised to distinguish among the three dimensions of delivery service quality—personal contact quality, customization quality, and operational quality—rather than treating delivery service quality as a single construct. Furthermore, terminology was standardized throughout the manuscript to improve conceptual consistency and readability.

The Introduction, Literature Review, Results and Discussion, Conclusions, and Implications sections were substantially revised to improve coherence and better articulate the study’s theoretical and practical contributions. We also added clarifications regarding the study context, ethics approval, participant consent procedures, common method bias assessment, data availability, and study limitations.

A detailed point-by-point response to all reviewer comments has been provided in the accompanying “Response to Reviewers” document.

We confirm that all authors have reviewed and approved the revised manuscript and agree with its submission to the journal. We further confirm that all authors approved the public sharing of the research data through the Figshare repository. The data supporting the findings of this study are openly available at https://doi.org/10.6084/m9.figshare.32587425.

We also confirm that the funder had no role in the study design, data analysis, interpretation of results, decision to publish, or preparation of the manuscript, except where explicitly disclosed in the manuscript.

We sincerely appreciate the opportunity to revise our work and hope that the revisions adequately address the reviewers’ concerns. We respectfully submit the revised manuscript for your further consideration and look forward to your response.

Sincerely,

Attachment Submitted filename: RESPONSE TO REVIEWER_2.docx

# Peer review: Decision Letter 2

**DOI:** 10.1371/journal.pone.0359308.r005

**Authors:** Cheong Kim

30 Jul 2026

Dear Dr. Manansala,

Thank you for submitting your manuscript to PLOS ONE. After careful consideration, we feel that it has merit but does not fully meet PLOS ONE’s publication criteria as it currently stands. Therefore, we invite you to submit a revised version of the manuscript that addresses the points raised during the review process.

Please submit your revised manuscript by Sep 13 2026 11:59PM. If you will need more time than this to complete your revisions, please reply to this message or contact the journal office at plosone@plos.org. When you're ready to submit your revision, log on to https://www.editorialmanager.com/pone/ and select the 'Submissions Needing Revision' folder to locate your manuscript file.

- A letter that responds to each point raised by the academic editor and reviewer(s). You should upload this letter as a separate file labeled 'Response to Reviewers'.
- A marked-up copy of your manuscript that highlights changes made to the original version. You should upload this as a separate file labeled 'Revised Manuscript with Track Changes'.
- An unmarked version of your revised paper without tracked changes. You should upload this as a separate file labeled 'Manuscript'.

If you would like to make changes to your financial disclosure, please include your updated statement in your cover letter. Guidelines for resubmitting your figure files are available below the reviewer comments at the end of this letter.

If applicable, we recommend that you deposit your laboratory protocols in protocols.io to enhance the reproducibility of your results. Protocols.io assigns your protocol its own identifier (DOI) so that it can be cited independently in the future. For instructions see: https://journals.plos.org/plosone/s/submission-guidelines#loc-laboratory-protocols. Additionally, PLOS ONE offers an option for publishing peer-reviewed Lab Protocol articles, which describe protocols hosted on protocols.io. Read more information on sharing protocols at https://plos.org/protocols?utm_medium=editorial-email&utm_source=authorletters&utm_campaign=protocols.

As the corresponding author, your ORCID iD is verified in the submission system and will appear in the published article. PLOS supports the use of ORCID, and we encourage all coauthors to register for an ORCID iD and use it as well. Please encourage your coauthors to verify their ORCID iD within the submission system before final acceptance, as unverified ORCID iDs will not appear in the published article. *Only* the individual author can complete the verification step; PLOS staff *cannot* verify ORCID iDs on behalf of authors.

We look forward to receiving your revised manuscript.

Kind regards,

Cheong Kim

Academic Editor

PLOS One

Journal Requirements:

If the reviewer comments include a recommendation to cite specific previously published works, please review and evaluate these publications to determine whether they are relevant and should be cited. There is no requirement to cite these works unless the editor has indicated otherwise.

[Note: HTML markup is below. Please do not edit.]

Reviewers' comments:

Reviewer's Responses to Questions

**Comments to the Author**

Reviewer #3: (No Response)

**********

2. Is the manuscript technically sound, and do the data support the conclusions?

Reviewer #3: Partly

**********

3. Has the statistical analysis been performed appropriately and rigorously?

Reviewer #3: No

**********

4. Have the authors made all data underlying the findings in their manuscript fully available?

The PLOS Data policy

Reviewer #3: Yes

**********

5. Is the manuscript presented in an intelligible fashion and written in standard English?

Reviewer #3: Yes

**********

Reviewer #3: Thank you for the opportunity to review this revised manuscript. The authors have made efforts to improve the manuscript. However, to improve the study's overall presentation and scientific rigor, several topics still need clarification and revision.

1.Introduction and Literature Review: The research gap and the study's significance are insufficiently articulated. The authors are encouraged to include a summary at the end of the Introduction or Literature Review that clearly highlights the research gap, the study's significance, and its contribution to the existing literature.

2.Conceptual Framework and Hypotheses: The presentation of the hypotheses and the conceptual framework could be improved to enhance clarity and consistency. Specifically, the terminology used to describe the same construct is not consistent across the hypotheses. For example, Hypotheses 3 and 4 refer to "last-mile delivery service quality", whereas Hypotheses 7–10 use "delivery service quality" when referring to the same construct. The authors are encouraged to use consistent terminology throughout the manuscript to avoid potential confusion. In addition, the conceptual framework does not indicate the corresponding hypothesis numbers (e.g., H1–H10). Including these labels would improve readability and help readers clearly link the conceptual model with the proposed hypotheses. Furthermore, the manuscript proposes separate hypotheses for customer participation behavior and customer citizenship behavior, suggesting that these represent distinct relationships. However, the conceptual framework illustrates these relationships using a shared path, making it difficult to distinguish the individual hypothesized effects. The conceptual framework should therefore be revised to accurately reflect the proposed hypotheses and provide a clearer visual representation of the research model. Moreover, the conceptual framework would benefit from greater consistency in the presentation of construct labels. Most constructs are presented using both their full names and abbreviations (e.g., CPB and DSQ). However, Customer Citizenship Behavior does not include its abbreviation in the framework. For consistency and ease of interpretation, the authors are encouraged to present abbreviations for all constructs uniformly.

3.Methodology and Participant Characteristics: The reporting of participants' demographic characteristics could be improved for completeness and consistency. In the demographic table, the total number of respondents is reported only for gender, whereas the totals for other variables (e.g., age, education, and income) are not presented. Reporting the frequencies and totals consistently across all demographic variables would improve the clarity of the sample description. Furthermore, in the manuscript (around Line 321), the authors state that the demographic information includes age, gender, education, income, and region. However, the demographic table does not report region, nor could it be identified elsewhere in the Results section. The authors should clarify whether regional information was collected and, if so, where these results are reported. If not, the description in the manuscript should be revised accordingly.

4.Ethical Approval: The manuscript states that ethical approval was obtained from De La Salle University. If available, the authors are encouraged to report the approval or reference number issued by the institutional ethics committee to improve transparency and facilitate verification.

5.Results Presentation: Descriptive results: The presentation of the descriptive statistics could be improved. Table 3 currently reports item-level descriptive statistics, making it difficult to obtain an overall understanding of each construct. The authors may consider presenting the descriptive statistics at the construct (variable) level or organizing the results by construct to improve readability and facilitate interpretation.

6.Measurement Model Reporting: The measurement model is presented in the tables, including factor loadings, Cronbach's alpha, composite reliability, AVE, and discriminant validity. However, the accompanying text provides only a general description of these results. The authors are encouraged to briefly summarize the key findings by referring to the reported values in the tables and supporting the evaluation criteria with appropriate methodological references (eg; Hair et al. 2022/2021/2019 or Fornell & Larcker, 1981). This would strengthen the transparency and completeness of the reporting.

7.Table Presentation: The presentation of Tables 6 and 7 could be improved by clearly defining all abbreviations used (e.g., construct abbreviations) in the table notes or captions. Providing the full names of the constructs would improve readability and help readers interpret the results without having to refer back to previous sections.

8.Hypothesis Development and Results Reporting: Reporting of hypothesis testing requires further clarification to ensure consistency among the proposed hypotheses, analytical approach, and reported results. For example, Hypotheses H3 and H4 propose relationships at the construct level (i.e., CPB →DSQ and CCB → DSQ). However, the corresponding analyses are reported at the dimensional level (e.g., CPB → PCQ, CPB → CQ, CPB → OC, and similarly for CCB), rather than at the construct level described in the hypotheses. A similar issue is observed in the mediation analysis (H5 and H6). The hypotheses are presented at the construct level (e.g., CPB/CCB → DSQ → Customer Satisfaction), whereas the reported mediation results appear to have been based on the DSQ dimensions. Consequently, it is difficult to determine whether the hypotheses were tested as originally proposed or whether the analyses were performed at a different level of the model. The authors should clarify the analytical strategy and ensure consistency between the conceptual framework, hypothesis development, statistical analyses, and results reporting. If the analyses are intended to be conducted at the dimensional level, the hypotheses should be reformulated accordingly (e.g., H3a–H3c, H4a–H4c, and corresponding mediation hypotheses). Alternatively, if the hypotheses are proposed at the construct level, the presentation of the analyses should be aligned with the stated hypotheses.

9.Consistency in Mediation/Moderation Terminology: Reporting of mediation and moderation analyses requires further clarification. In the manuscript (around Line 552), the authors refer to the results as the “mediating effects of digital accessibility.” However, based on the proposed conceptual framework and hypotheses, digital accessibility appears to be examined as a moderating variable. The authors should verify and revise the terminology to ensure consistency between the conceptual model, hypotheses, analytical approach, and interpretation of results. If digital accessibility is intended to represent a moderator, the results section should refer to moderating effects rather than mediating effects. In addition, the relevant sections of the manuscript (e.g., hypothesis development, conceptual framework, and discussion) should be reviewed and revised to maintain consistency throughout.

Overall, this manuscript addresses a relevant topic in the context of co-creation customer satisfaction and last-mile delivery services.The study has the potential to contribute to the existing literature; however, several aspects require further clarification and improvement, particularly regarding the alignment between the conceptual framework, hypothesis development, analytical approach, and results reporting. The authors are encouraged to carefully revise the manuscript to improve conceptual clarity, methodological transparency, and consistency in the interpretation of findings. Addressing these issues would strengthen the scientific rigor and overall quality of the manuscript.

**********

what does this mean?). If published, this will include your full peer review and any attached files.

If you choose “no”, your identity will remain anonymous but your review may still be made public.

**Do you want your identity to be public for this peer review?** For information about this choice, including consent withdrawal, please see our Privacy Policy

Reviewer #3: No

**********

[NOTE: If reviewer comments were submitted as an attachment file, they will be attached to this email and accessible via the submission site. Please log into your account, locate the manuscript record, and check for the action link "View Attachments". If this link does not appear, there are no attachment files.]

To ensure your figures meet our technical requirements, please review our figure guidelines: https://journals.plos.org/plosone/s/figures

You may also use PLOS’s free figure tool, NAAS, to help you prepare publication quality figures: https://journals.plos.org/plosone/s/figures#loc-tools-for-figure-preparation.

NAAS will assess whether your figures meet our technical requirements by comparing each figure against our figure specifications.

# Author comment: Author response to Decision Letter 3

**DOI:** 10.1371/journal.pone.0359308.r006

19 Aug 2026

Response to Reviewer #3

We sincerely thank Reviewer #3 for the careful and constructive assessment of the revised manuscript. The comments have helped us further improve the manuscript's conceptual clarity, methodological transparency, terminological consistency, and alignment between the conceptual framework, hypotheses, analyses, results, and conclusions. We have revised the manuscript accordingly while retaining the original study design, dataset, and analytical approach. The responses below identify the revisions made and the corresponding sections of the manuscript.

General Revision Note

Throughout the revised manuscript, Delivery Service Quality (DSQ) is treated as a conceptual multidimensional construct comprising Personal Contact Quality (PCQ), Customization Quality (CQ), and Operational Quality (OQ). The empirical analyses examine PCQ, CQ, and OQ separately. The hypothesis numbering and discussion have therefore been aligned with the dimensional analyses reported in Tables 8–10.

Comment 1. Introduction and Literature Review

Reviewer Comment:

The research gap and the study's significance are insufficiently articulated. The authors are encouraged to include a summary at the end of the Introduction or Literature Review that clearly highlights the research gap, the study's significance, and its contribution to the existing literature.

Response:

Thank you for this important observation. We agreed that the previous presentation identified several gaps without sufficiently explaining how they formed a coherent research problem. We therefore consolidated the gaps into three interconnected gaps that directly correspond to the empirical model.

Revision Made:

The Introduction was revised to present three connected gaps: (1) the distinct effects of CPB and CCB on customer satisfaction in last-mile delivery; (2) the separate mediating roles of PCQ, CQ, and OQ; and (3) the selective moderating role of DA. We also added an explicit explanation of why these gaps matter theoretically and practically, emphasizing that last-mile delivery increasingly depends on interactions among customers, providers, and digital platforms. The concluding paragraphs of the Introduction now explicitly state the study's theoretical and practical contributions.

Location:

Introduction – research gap, study significance, and contribution paragraphs.

Comment 2. Conceptual Framework and Hypotheses

Reviewer Comment:

The presentation of the hypotheses and conceptual framework could be improved. The terminology used to describe the same construct is not consistent. H3 and H4 refer to last-mile delivery service quality, whereas H7–H10 use delivery service quality. The framework should use consistent terminology, include H1–H10 labels, distinguish CPB and CCB paths, and present abbreviations consistently.

Response:

We appreciate this observation and have revised the conceptual presentation accordingly. The formal construct terminology is now standardized as Delivery Service Quality (DSQ), with PCQ, CQ, and OQ identified as its three dimensions. The phrase “last-mile delivery service quality” is retained only as contextual language and is no longer used as an alternative construct label.

Revision Made:

The conceptual framework has been revised to:

use DSQ consistently;

show PCQ, CQ, and OQ as the three DSQ dimensions;

distinguish CPB and CCB paths separately;

display hypothesis labels on the corresponding paths; and

present abbreviations consistently, including CCB and DA.

The hypothesis structure was also revised to H3a–H3c, H4a–H4c, H5a–H5c, H6a–H6c, H7a–H7c, and H8a–H8c to match the dimensional analyses.

Location:

Conceptual Framework and Hypotheses; revised research model and hypothesis development.

Comment 3. Methodology and Participant Characteristics

Reviewer Comment:

The demographic table should report frequencies and totals consistently. The manuscript also states that demographic information includes region, but region is not reported in the table or Results section. Please clarify whether regional information was collected.

Response:

Thank you. We reviewed the demographic reporting and revised the presentation to ensure that the sample description corresponds exactly to the information actually collected and reported. We have avoided retaining a demographic category that is not supported by the reported results.

Revision Made:

The demographic table was revised to report frequencies and percentages consistently across the reported demographic variables, with the total sample of 426 respondents clearly indicated. The manuscript's description of demographic information was also revised so that it does not imply the collection or reporting of regional information unless such information is actually presented in the results.

Location:

Methodology – Participant Characteristics / Table 2.

Comment 4. Ethical Approval

Reviewer Comment:

The manuscript states that ethical approval was obtained from De La Salle University. If available, the authors are encouraged to report the approval/reference number.

Response:

We agree that reporting the institutional ethics committee more specifically improves transparency. The ethics statement has therefore been clarified.

Revision Made:

The revised Ethics statement identifies the approving body as the De La Salle University–Dasmariñas Research Ethics Committee. The statement also clarifies that approval was obtained before data collection, informed consent was obtained electronically through the online survey, all participants were adults aged 18 years or older, no minors were involved, and no waiver of consent was granted. Where an institutional approval/reference number is available, it is reported in the revised statement.

Location:

Ethical Approval and Participant Consent.

Comment 5. Results Presentation – Descriptive Results

Reviewer Comment:

Table 3 currently reports item-level descriptive statistics, making it difficult to obtain an overall understanding of each construct. The authors may consider presenting descriptive statistics at the construct level or organizing the results by construct.

Response:

We appreciate this recommendation. We revised the presentation so that readers can more readily interpret the descriptive characteristics of the measured constructs without losing the underlying item-level information where it is needed for measurement assessment.

Revision Made:

The descriptive-results presentation was reorganized to improve construct-level readability. The narrative now emphasizes the overall descriptive characteristics of the constructs, while item-level information is retained where appropriate for measurement-model reporting.

Location:

Results – Descriptive Statistics / Table 3.

Comment 6. Measurement Model Reporting

Reviewer Comment:

The measurement model is presented in the tables, including factor loadings, Cronbach's alpha, composite reliability, AVE, and discriminant validity. The accompanying text provides only a general description. Please briefly summarize key findings and support evaluation criteria with methodological references such as Hair et al. or Fornell and Larcker.

Response:

We agree. The measurement-model discussion was expanded to interpret the reported values rather than merely directing readers to the tables.

Revision Made:

The revised measurement-model section now summarizes the observed ranges and findings for factor loadings, Cronbach's alpha, composite reliability, and AVE and explicitly relates them to established methodological criteria. Discriminant validity is also discussed using the appropriate assessment criteria and supported by methodological references, including Hair et al. and Fornell and Larcker where applicable.

Location:

Measurement Model – Tables 4–7 and accompanying discussion.

Comment 7. Table Presentation

Reviewer Comment:

Tables 6 and 7 should clearly define all abbreviations used, preferably in table notes or captions, and provide full construct names for readability.

Response:

We agree and have standardized the table notes so that readers can interpret the tables without repeatedly referring to earlier sections.

Revision Made:

Table notes and captions were revised to define the construct abbreviations consistently. The relevant notes identify CPB, CCB, DA, PCQ, CQ, OQ, and CS using their full construct names. This convention is applied consistently across the measurement and structural-model tables, including Tables 8–11.

Location:

Tables 6–11 – revised notes/captions.

Comment 8. Hypothesis Development and Results Reporting

Reviewer Comment:

H3 and H4 are proposed at the DSQ construct level, but the analyses are reported at the dimensional level (PCQ, CQ, OQ). The same issue occurs for H5 and H6. The authors should either analyze the aggregate construct or reformulate the hypotheses at the dimensional level.

Response:

We agree with the reviewer that the original wording created a mismatch between the hypotheses and the empirical analyses. Because the reported PLS-SEM model estimates PCQ, CQ, and OQ separately, we revised the hypotheses rather than introducing an unreported aggregate DSQ analysis.

Revision Made:

The hypotheses were reformulated as follows:

H3a–H3c: CPB → PCQ, CQ, and OQ;

H4a–H4c: CCB → PCQ, CQ, and OQ;

H5a–H5c: CPB → PCQ/CQ/OQ → CS; and

H6a–H6c: CCB → PCQ/CQ/OQ → CS.

The moderation hypotheses were similarly revised to H7a–H7c and H8a–H8c for the three DSQ dimensions, while H9 and H10 retain the moderation paths to customer satisfaction.

The Model Specification section now provides equations corresponding to these paths. Tables 8–10 and the Results, Discussion, and Conclusions have been revised to use the same dimensional structure.

Location:

Model Specification; Hypothesis Development; Tables 8–10; Results and Discussion; Conclusions.

Comment 9. Consistency in Mediation/Moderation Terminology

Reviewer Comment:

The manuscript refers to digital accessibility as having “mediating effects,” although it is proposed and analyzed as a moderator. Please revise the terminology consistently.

Response:

Thank you for identifying this terminology error. We agree that digital accessibility is a moderator in the conceptual and statistical model and should not be described as a mediator.

Revision Made:

All relevant sections were reviewed and revised. Digital accessibility is now consistently described as a moderating variable, and the results are reported as “moderating effects” or “interaction effects.” The manuscript no longer refers to DA as a mediator. The moderation discussion also explicitly distinguishes the significant interaction effects from the non-significant moderation effects on customer satisfaction.

Location:

Conceptual Framework; Model Specification; Table 10 Discussion; Conclusions and Implications.

Additional Cross-Manuscript Revisions

In addition to the specific revisions above, we conducted a cross-manuscript consistency review.

Terminology:

The manuscript consistently uses Delivery Service Quality (DSQ) as the conceptual multidimensional construct and PCQ, CQ, and OQ as its separately analyzed dimensions.

Direct Effects:

Table 8 and its discussion now report H1–H4 at the level actually estimated, with H3a–H3c and H4a–H4c explicitly identified.

Mediation:

Table 9 is now focused on specific indirect effects. The discussion reports H5a–H5c and H6a–H6c separately rather than treating DSQ as a single mediator.

Moderation:

Table 10 and its discussion report H7a–H7c, H8a–H8c, H9, and H10 separately. DA is consistently treated as a moderator.

Model Fitness:

Table 11 is interpreted at the level of PCQ, CQ, OQ, and CS, rather than implying a single aggregate DSQ R² or Q².

Conclusions and Implications:

The conclusions and implications were revised to reflect the exact supported and unsupported hypotheses and the dimensional mediation and moderation results.

Dark-Side Perspective:

The Introduction and interpretation now frame co-creation as potentially beneficial but contingent on whether customer engagement is translated into service-quality outcomes, avoiding claims that co-creation is universally beneficial.

Scope of Claims:

Claims concerning unmeasured variables such as organizational architectures, trust, loyalty, fairness, convenience, effort-benefit balance, and independent operational improvements have been removed or reframed as contextual considerations rather than tested variables.

In closing, we again thank Reviewer #3 for the constructive recommendations. The revisions have been made with particular attention to ensuring that the conceptual framework, hypotheses, statistical model, tables, interpretation, and conclusions describe the same empirical relationships. We believe these changes substantially improve the manuscript's conceptual coherence, methodological transparency, and readability while preserving the study's original design and contribution.

Attachment Submitted filename: Response_to_Reviewer_3_Revised_Manuscript.docx

# Peer review: Decision Letter 3

**DOI:** 10.1371/journal.pone.0359308.r007

**Authors:** Cheong Kim

13 Sep 2026

VALUE CO-CREATION AND CUSTOMER SATISFACTION IN LAST-MILE DELIVERY SERVICES: THE ROLES OF DIGITAL ACCESSIBILITY AND DELIVERY SERVICE QUALITY

PONE-D-25-66376R3

Dear Dr. Manansala,

We’re pleased to inform you that your manuscript has been judged scientifically suitable for publication and will be formally accepted for publication once it meets all outstanding technical requirements.

Within one week, you’ll receive an e-mail detailing the required amendments. When these have been addressed, you’ll receive a formal acceptance letter and your manuscript will be scheduled for publication.

An invoice will be generated when your article is formally accepted. Please note, if your institution has a publishing partnership with PLOS and your article meets the relevant criteria, all or part of your publication costs will be covered. Please make sure your user information is up-to-date by logging into Editorial Manager at Editorial Manager® and clicking the ‘Update My Information' link at the top of the page. For questions related to billing, please contact billing support.

If your institution or institutions have a press office, please notify them about your upcoming paper to help maximize its impact. If they’ll be preparing press materials, please inform our press team as soon as possible -- no later than 48 hours after receiving the formal acceptance. Your manuscript will remain under strict press embargo until 2 pm Eastern Time on the date of publication. For more information, please contact onepress@plos.org.

Kind regards,

Cheong Kim

Academic Editor

PLOS One

Reviewers' comments:

Reviewer's Responses to Questions

**Comments to the Author**

Reviewer #3: All comments have been addressed

**********

2. Is the manuscript technically sound, and do the data support the conclusions?

Reviewer #3: Yes

**********

3. Has the statistical analysis been performed appropriately and rigorously?

Reviewer #3: Yes

**********

4. Have the authors made all data underlying the findings in their manuscript fully available?

The PLOS Data policy

Reviewer #3: Yes

**********

5. Is the manuscript presented in an intelligible fashion and written in standard English?

Reviewer #3: Yes

**********

Reviewer #3: Thank you for carefully addressing the previous comments. The manuscript has been substantially improved, and I am generally satisfied with the revisions. I have only one minor suggestion regarding the interpretation of Harman’s single-factor test, particularly the statement linking it to construct reliability and PLS-SEM validity. Please consider moderating this statement accordingly. Overall, I have no further major concerns.

**********

what does this mean?). If published, this will include your full peer review and any attached files.

If you choose “no”, your identity will remain anonymous but your review may still be made public.

**Do you want your identity to be public for this peer review?** For information about this choice, including consent withdrawal, please see our Privacy Policy

Reviewer #3: No

**********

# Editor report: Acceptance letter

**DOI:** 10.1371/journal.pone.0359308.r008

**Authors:** Cheong Kim

PONE-D-25-66376R3

PLOS One

Dear Dr. Manansala,

I'm pleased to inform you that your manuscript has been deemed suitable for publication in PLOS One. Congratulations! Your manuscript is now being handed over to our production team.

At this stage, our production department will prepare your paper for publication. This includes ensuring the following:

* All references, tables, and figures are properly cited

* All relevant supporting information is included in the manuscript submission,

* There are no issues that prevent the paper from being properly typeset

You will receive further instructions from the production team, including instructions on how to review your proof when it is ready. Please keep in mind that we are working through a large volume of accepted articles, so please give us a few days to review your paper and let you know the next and final steps.

Lastly, if your institution or institutions have a press office, please let them know about your upcoming paper now to help maximize its impact. If they'll be preparing press materials, please inform our press team within the next 48 hours. Your manuscript will remain under strict press embargo until 2 pm Eastern Time on the date of publication. For more information, please contact onepress@plos.org.

You will receive an invoice from PLOS for your publication fee after your manuscript has reached the completed accept phase. If you receive an email requesting payment before acceptance or for any other service, this may be a phishing scheme. Learn how to identify phishing emails and protect your accounts at https://explore.plos.org/phishing.

If we can help with anything else, please email us at customercare@plos.org.

Thank you for submitting your work to PLOS One and supporting open access.

Kind regards,

PLOS One Editorial Office Staff

on behalf of

Dr. Cheong Kim

Academic Editor

PLOS One
